# A Comprehensive Study on Gravlax: A Multidimensional Evaluation of Gravlax Produced from Different Fish Species and Herbs

**DOI:** 10.3390/foods14142465

**Published:** 2025-07-14

**Authors:** Can Okan Altan

**Affiliations:** 1 Department of Seafood Processing Technology, Fisheries Faculty, Sinop University, Sinop 57000, Türkiye; okanaltan@sinop.edu.tr; Tel.: +90-5334746910; 2 Scientific and Technological Research Application and Research Center, Sinop University, Sinop 57000, Türkiye

**Keywords:** gravlax, fish, tub gurnard, garfish, sensory analyses, minerals and heavy metals, dill, sage, mint, basil

## Abstract

In this study, gravlax, a niche Scandinavian delicacy, was comprehensively investigated by producing it with combinations of two different fish species (tub gurnard (*Chelidonichthys lucerna* Linnaeus, 1758) and garfish (*Belone belone* Linnaeus, 1761)) and five herbs (dill (*Anethum graveolens* Linnaeus, 1753), sage (*Salvia officinalis* Linnaeus, 1753), mint (*Mentha piperita* Linnaeus, 1753), sweet (*Ocimum basilicum* Linnaeus, 1754), and purple basil (*Ocimum basilicum* var. *purpurascens* Bentham, 1830)). The nutritional composition, amino acids, color parameters, mineral substances, and heavy metal content, as well as physical characteristics, texture profile analysis, and extensive sensory analyses, were conducted, and the results were thoroughly evaluated using multivariate statistical methods. The influence of using different herbs on nutritional composition was found to be significant in gravlax made from both fish species (*p* < 0.05). Sensory analyses revealed that panelists identified mint as enhancing aroma and umami sensations, while dill improved overall acceptance. Gravlax with sage exhibited softer textures, but lower general acceptance due to perceived high saltiness. Color analyses revealed that purple basil transferred distinct pigments, causing darkening, whereas sweet basil had a brightening effect. Amino acid analyses revealed higher umami and sweet-tasting amino acids in herb-free gravlax, whereas proteolytic activity appeared to slow down in herb-containing gravlax samples.

## 1. Introduction

Gravlax is a gourmet delicacy of Scandinavian origin, traditionally prepared from salmon. The production process involves curing fish with a mixture of herbs, most commonly dill, salt, and brown sugar, for a period of 36 to 72 h [1,2].

The word “*gravlax*” originates from the northern German terms “*gravad*” (buried) and “lax/laks” (salmon), and as stated in the Swedish Academy Dictionary (Ordbok öfver svenska språket), gravlaks, which means “*eingegrabener lachs*” or “*buried salmon*”, referred historically to a fish product obtained by rubbing salmon with salt and then burying it underground, a method used to preserve salmon before refrigeration was available [3]. The Oxford Companion to Food [4] states that gravlax dates back to Scandinavian history as early as 1348, attributing the name to a man from Jämtland named “Olafuer Gravlax”.

The same source describes the more recent preparation of gravlax as follows: “In modern times, burying fish is no longer necessary to prepare gravlax. The fish is cleaned, scaled, split lengthwise, and deboned. A fillet is placed skin-side down into a suitable container and seasoned with fresh dill, coarse salt, a bit of sugar, and black pepper. Another fillet of the same size is placed on top, skin-side up, followed by a board and a heavy weight. This setup is left in a cool place for approximately three days, with the fish being flipped every 12 h and basted with the juices it releases. Traditionally, gravlax preparation is considered among men’s household chores. Before serving, gravlax is drained and brushed clean. It is thinly sliced and commonly served as an appetizer with a special mild mustard sauce, fresh dill, or potato salad [4].”

Gravlax contains significantly less salt compared to other salted fish products, making it a more suitable option in terms of sodium intake. Additionally, as a minimally processed food, gravlax retains a higher nutritional value compared to heavily processed or cured fish products. Due to its minimal processing, the nutritional quality of gravlax closely resembles fresh fish meat. Rich in omega-3 fatty acids and very low in saturated fats, this gourmet product supports cardiovascular health, while its high protein content aids muscle recovery and helps maintain muscle tissue. Moreover, gravlax is also a source of essential vitamins and minerals such as B vitamins, vitamin D, and selenium [5]. A traditionally prepared serving of gravlax (57 g/2 oz) contains approximately 12 g protein, 6 g unsaturated fats, 2 g saturated fats, 6 g carbohydrates, 211 mg potassium, 600 mg sodium, and 6.3 mg calcium [6].

In terms of texture, gravlax occupies an intermediate position between salted and marinated fish products, displaying characteristics that more closely resemble the flesh of fresh fish compared to those produced by conventional salting and marinating methods. The growing popularity of gravlax in recent years has prompted increased research into its production, including the use of various fish species and herb-based ingredients [1,7]. While gravlax is typically associated with salmon, successful production has also been reported with other species, such as mackerel, bonito, whitefish, rainbow trout, and herring, with promising results [2,8,9,10,11].

Garfish (*Belone belone*) is a migratory pelagic fish species inhabiting the coastal waters of Europe and North Africa, as well as the North Sea, Black Sea, the Mediterranean coasts of France, Spain, Portugal, Morocco, and the North Atlantic [12]. Tub gurnard (*Chelidonichthys lucerna*) is a nektobenthic fish species distributed throughout the Eastern Atlantic from Norway to Senegal, as well as the Mediterranean and Black Sea regions [13]. Both species are commonly found in the Black Sea.

In this study, these two fish species were selected for gravlax production due to their unique characteristics and the fact that they have not been previously investigated for use in gravlax to date. The two species exhibit markedly contrasting features. For instance, tub gurnard is characterized by thick fillets and white flesh, whereas garfish possesses dark flesh and extremely thin fillets. Furthermore, their nutritional compositions, including amino acid and mineral contents, differ considerably. The investigation of these contrasting species under identical conditions aims to elucidate potential outcomes in gravlax production. Additionally, both species are readily available and affordable in the Black Sea throughout nearly all seasons.

Various herbs are utilized as flavoring agents in the development of gastronomic products [2]. Dill is traditionally used in the preparation of gravlax. However, research on the use of alternative herb species in gravlax production remains quite limited [2,11]. In addition to dill, this study selected herbs that are both readily accessible and familiar in terms of flavor in daily life. Observations conducted in local markets and supermarkets indicated that dill, mint, basil, and purple basil were suitable candidates for gravlax experiments. Although sage is not a widely prevalent herb in the region, it was included in the study due to its well-documented health benefits and its potential to impart distinctive physical, chemical, and sensory properties to fish tissue. Fresh sage was sourced locally and employed in the gravlax production process.

This study aims to address the need for greater innovation and diversity in gravlax products by systematically exploring the combined use of different fish species and aromatic herbs. Another key objective is to introduce new alternatives to seafood cuisine, particularly targeting the preferences of the younger generation, who increasingly favor minimally or semi-processed seafood products. The central hypothesis is that both the type of fish and the selection of herbs, as well as their interactions, play a decisive role in shaping the nutritional profile, physicochemical characteristics, and sensory appeal of gravlax. By evaluating various fish–herb pairings, the research examines not only the individual contributions of each component but also whether synergistic effects can enhance product quality beyond what either component achieves alone. Through comprehensive physical, chemical, and sensory analyses, this study seeks to generate new insights that may guide the development of healthier, more appealing semi-processed seafood products and foster increased consumer awareness of gravlax as a nutritious option.

## 2. Materials and Methods

### 2.1. Materials

**Figure 1:**
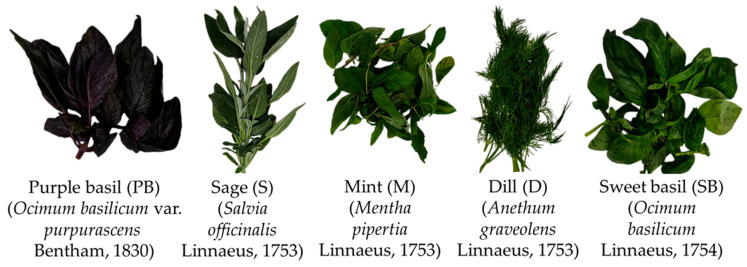
The original images of the herbs used in the research.

The fish species, garfish (*Belone belone* Linnaeus, 1761) and tub gurnard (*Chelidonichthys lucerna* Linnaeus, 1758), were purchased in December 2024 from the Sinop Fish Market (Sinop, Black Sea, Türkiye) less than two hours after being caught, ensuring optimal freshness. Immediately following purchase, the fish were transported within one hour in ice-filled polystyrene boxes to the Seafood Processing Laboratory at the Faculty of Fisheries, Sinop University, TR. The herbs used in the research, including dill (*Anethum graveolens* Linnaeus, 1753), sage (*Salvia officinalis* Linnaeus, 1753), mint (*Mentha piperita* Linnaeus, 1753), sweet basil (*Ocimum basilicum* Linnaeus, 1754), and purple basil (*Ocimum basilicum* var. *purpurascens* Bentham, 1830), were sourced from the Sinop Local Market (Sinop, Black Sea, Türkiye), where they are freshly harvested and sold daily (Figure 1). Brown sugar, black pepper, coriander, and sea salt used in gravlax production were all obtained from the Migros supermarket chain (Istanbul, Türkiye).

Prior to processing, the length and weight of the fish were measured. The average length and weight of garfish were determined to be 35.16 ± 0.46 cm and 44.90 ± 2.26 g, respectively (*n* = 108). For the tub gurnard, the average length was 47.87 ± 0.41 cm, and the average weight was 1126.60 ± 30.84 g (*n* = 15).

**Figure 2:**
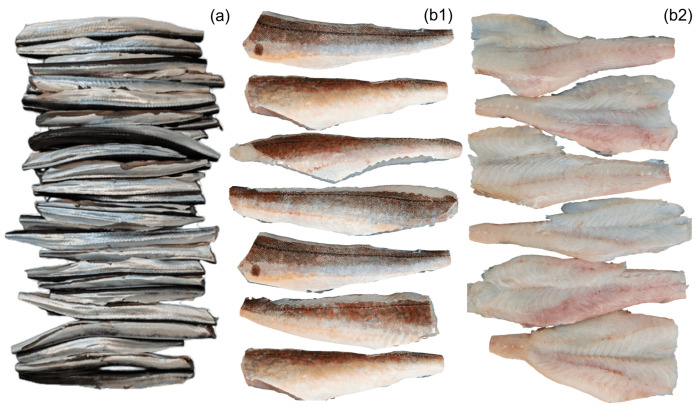
Deboned fish fillets. (**a**) Deboned garfish; (**b1**) outer side of tub gurnard fillets; (**b2**) inner side of tub gurnard fillets.

After the internal organs of the fish were removed, the bones of the garfish were carefully extracted, while the tub gurnard was filleted. Any remaining bones were removed using tweezers. The fillet yield was calculated as 58.36% for garfish and 41.32% for tub gurnard. For the calculation of fillet thickness, the thickest and thinnest points of the tub gurnard fillets were measured on a plane with a caliper, while for garfish fillets, the measurement was taken directly from the center. Accordingly, the average fillet thickness was determined as 0.57 ± 0.08 cm (*n* = 50) for garfish and 1.39 ± 0.14 cm (*n* = 30) for tub gurnard. Then, the fillets were immersed in a 5% ice-cold water saline solution for 15 min to remove blood and other residues. Subsequently, they were transferred to non-saline ice-cold water for 5 min and then drained using sieves for 15 min to remove excess water. Images of the cleaned fish fillets are presented in Figure 2.

All herbs were first soaked in 5 L of cold water containing sodium carbonate at a concentration of 10 g/L for 1 h. This was followed by immersion in 5 L of cold water containing 0.5% white vinegar for 15 min. The herbs were then thoroughly rinsed under running tap water to remove physical residues. After draining for 30 min in a sieve, the herbs were placed on hygienic, cotton-based towels and dried thoroughly.

### 2.2. Methods

#### 2.2.1. Gravlax Production and Grouping

For gravlax production, the formulation previously developed by Altan et al. [2] was employed. The formulation is as follows:

For each kg of fillet: 200 g sea salt, 200 g brown sugar, one tablespoon of coriander, one tablespoon of black pepper, and 200 g of aromatic herbs.

**Figure 3:**
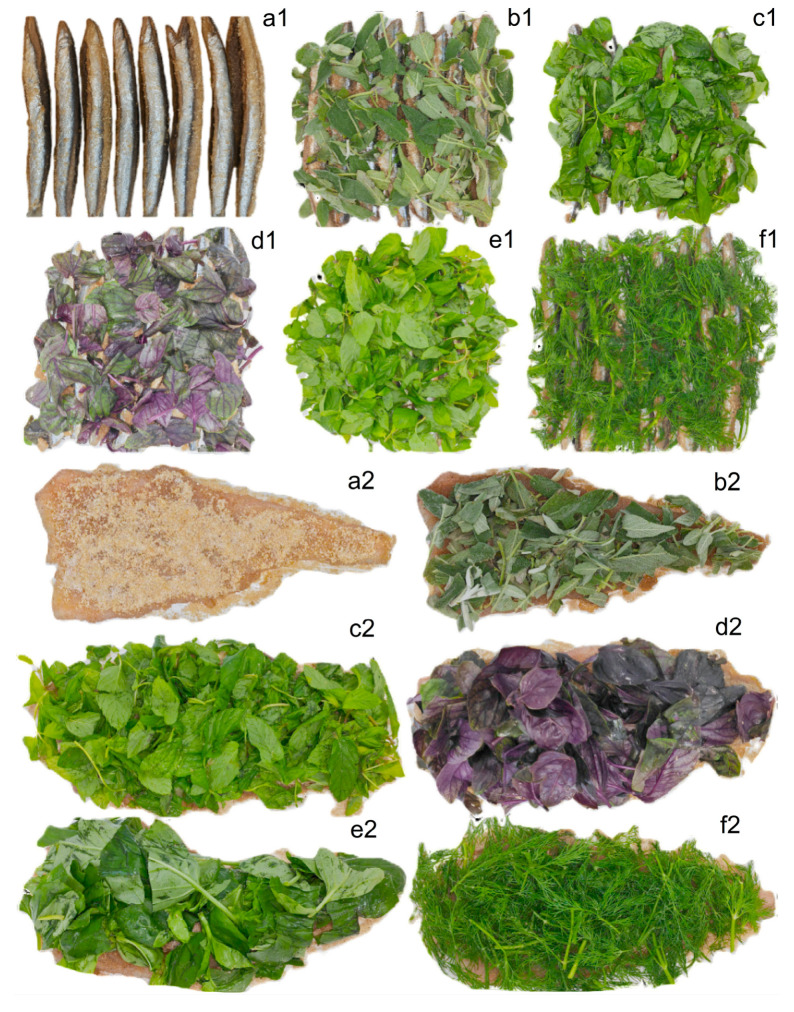
Gravlax preparation stage. The letters in the upper right corners of the images represent the gravlax groups based on the type of herb used: (**a**) no herbs, (**b**) sage, (**c**) mint, (**d**) purple basil, (**e**) sweet basil, and (**f**) dill. The numbers indicate the fish species, with (**1**) referring to garfish and (**2**) referring to tub gurnard.

The measured amounts of sea salt, brown sugar, coriander, and black pepper were placed in a mixing bowl. All dry ingredients were homogenized using an electric mixer for 2 min. Plastic wrap was laid out on a cutting board, and half of the corresponding amount of herbs (according to fillet weight) was evenly spread over the surface. Next, half of the premixed dry ingredients (per 1 kg of fillet) were uniformly distributed on top of the herbs. Skinless tub gurnard fillets were then placed on this layer, after which the remaining half of the dry mixture and herbs were evenly spread over the top side of the fillets (Figure 3). The fillets were then tightly wrapped in plastic film to form rolls, leaving both ends open to prevent the accumulation of exuded liquid within the roll.

The same procedure was applied to garfish fillets; however, due to the thin structure of garfish fillets and their unsuitability for skin removal, the fillets were processed with the skin intact. The prepared gravlax rolls were placed at a 45° angle in perforated containers, ensuring that any liquid exuded from the fish did not come into contact with the fillets during refrigeration. The gravlax rolls were turned every 6 h to promote uniform drainage of fluid. After curing at 3 ± 1 °C for 42 h in the refrigerator, the gravlax rolls were removed from the plastic wrap, and any wilted herbs were discarded from the fillet surfaces.

The grouping names and abbreviations for the groups are presented in Table 1.

#### 2.2.2. Analyses

##### Color Analyses

Color parameters (L*, a*, b*) were determined using a Konica Minolta CR-400 colorimeter (light projection tube model: A33a, Tokyo, Japan), set to a 2° standard observer and equipped with an 8.0 mm closed-cone aperture. Measurements were taken at twelve distinct locations (*n* = 12) on each gravlax fillet, ensuring that each measurement point was at least 3 cm apart to provide a representative assessment of the whole fillet.

The CIE Lab* color space, as recommended by the Commission Internationale de l’Eclairage (CIE) [14], was used for all analyses. In this system, L* indicates lightness (ranging from 0 for black to 100 for white), a* denotes the position between red and green, and b* represents the position between yellow and blue [14,15].

From these primary color coordinates, several derived color indices were calculated: Hue angle (1) (*h°*), chroma (2) (C*), the total color difference (3, 4) (*ΔE*), whiteness index (5) (*WI*), and yellowing index (6) (*YI*) [16]. C* indicates color saturation, while *h°* qualitatively describes the dominant color. The *WI* and *YI* were utilized to monitor changes in whiteness and yellowness, respectively, which are particularly relevant in the context of drying or product degradation [17]. *ΔE* quantifies the overall color variation between the experimental samples and reference samples. Specifically, *ΔE*_1_ reflects the difference between gravlax samples and untreated fresh fish fillets, whereas *ΔE*_2_ compares gravlax samples with fresh herbs.

All calculations were based on the following formulas using L*, a*, and b* values [16,18]:

(1) $$h^{{}^{\circ}}={tan}^{-1}(b^{*}/a^{*}),$$

(2) $$C^{*}=\sqrt{a^{{*}^{2}}+b^{{*}^{2}}},$$

(3) $${\Delta E}_{1}=\sqrt{{\left({a^{*}}_{gravlax}-{a^{*}}_{freshfillet}\right)}^{2}+{\left({b^{*}}_{gravlax}-{b^{*}}_{freshfillet}\right)}^{2}+{\left({L^{*}}_{gravlax}-{L^{*}}_{freshfillet}\right)}^{2}},$$

(4) $${\Delta E}_{2}=\sqrt{{\left({a^{*}}_{gravlax}-{a^{*}}_{freshherb}\right)}^{2}+{\left({b^{*}}_{gravlax}-{b^{*}}_{freshherb}\right)}^{2}+{\left({L^{*}}_{gravlax}-{L^{*}}_{fershherb}\right)}^{2}},$$

(5) $$WI=\sqrt{{\left(100-L^{*}\right)}^{2}+a^{{*}^{2}}+b^{{*}^{2}}},$$

(6) $$YI=142.86\times b^{*}/L^{*}.$$

##### pH, Water Activity (aw), and Salt Analyses

pH measurements were performed using a pH meter (Model: HI 98100, Hanna Instruments Inc., Smithfield, RI, USA) at 20 °C (*n* = 6), in accordance with Boland et al. [19]. Water activity (aw) was determined using a Lab Swift water activity device (Novasina AG, Lachen, Switzerland) at 20.4 °C (*n* = 6). Salt content (%) was determined using Mohr’s method [20] (*n* = 4).

##### Nutritional Analyses

Protein (P), ash (A), moisture (M), and lipid (L) analyses were performed according to AOAC (ref: 925.04, 938.08, 954.01, and 991.36) [21] . The carbohydrate content was calculated by subtracting the sum of the four primary nutrient components from 100% (7) [22]. Energy values were calculated using the Atwater conversion factors (8) [22]. All proximate analyses were performed in duplicate, with each replicate consisting of three parallel samples (*n* = 6).

(7) $$Carbohydrate\left(\%\right)=100-\left(M+L+P+A\right),$$

(8) $$Energy\left(Kcal\right)=\left(lipid\times 9\right)+\left(protein\times 4\right)+\left(carbohydrate\times 4\right).$$

##### Amino Acid Analyses

Amino acid (AA) analyses were conducted at the Sinop University Central Laboratory Facility (SÜBİTAM, Sinop, Türkiye) using liquid chromatography-mass spectrometry (LC-MS/MS) following hydrolysis with 6 N HCl. The analyses were performed using the high-performance liquid chromatography (HPLC) pre-column derivatization method described by Ishida et al. [23] (*n* = 3).

##### Sensorial Analyses

According to Stone and Sidel [24], laboratory-based sensory tests typically include 25 to 50 participants per product. In the present study, each developed product was evaluated by all 56 panelists in accordance with the described protocol. The sensory evaluation panel consisted of 56 participants aged between 24 and 28, of whom 42 held at least a master’s degree in seafood processing technology, while an additional 14 panelists aged between 33 and 42 were faculty members with doctoral degrees from the same department. Panelists were selected based on their prior experience—specifically, participation in at least two sensory evaluation studies—and their willingness to consume gravlax products. This approach ensured a more objective and reliable assessment of the sensory profile. Considering the product’s distinctive nature and the required depth of analysis, experienced panelists capable of detailed and informed evaluations were preferred. Barton et al. [25] indicate that panelists’ sensory method knowledge significantly influences evaluation outcomes, suggesting that experienced panelists are suitable evaluators in cases of limited resources or sample availability. Consequently, the panel size was intentionally limited to 56, striking a balance between the need for assessment reliability and specialized insight. According to the regulations of the Human Research Ethics Committee of Sinop University, informed consent was clearly explained verbally to all panelists who volunteered to participate in the study, and their written consent was documented with signatures. The sensory evaluations were conducted at the Quality Control Laboratory of Sinop University, Faculty of Fisheries. Prior to the tastings, detailed information was provided to the panelists regarding the study, the herbs, and the sensory evaluation forms. Sensory analyses were carried out in two separate stages. In the first stage, before proceeding to the gravlax products, each herb was tasted individually in its plain form, and panelists were asked to rate them using the questions presented in Table S1 on a scale from 1 to 9. After each herb tasting, panelists were instructed to drink water and to wait at least 5 min before tasting the following sample. Once the last panelist completed the herb-tasting section, all panelists were asked to wait for 10 min, after which they were provided with the sensory evaluation form developed by combining and modifying the sensory analysis forms of Altan et al. [2] and Sallam et al. [26] to suit the product (Table S2). They were then asked to taste the gravlax products and score them on a 1 to 9 scale. However, for the final question on the table, “favorite gravlax,” the panelists were asked to indicate their only top three preferences among the ten different gravlax groups by marking “*x*” for “I liked it less, but it is still among my preferences,” “*xx*” for “I liked it, and I may want to consume it in the future,” and “*xxx*” for “I liked it very much, and I would definitely want to consume it again in the future.” For the calculation of scores for this question, the medians of “*x*” marks given to each group were calculated, normalized, and converted into percentage (%) scores for statistical analyses. Panelists were also advised to wait at least 5 min and drink water before proceeding to each product tasting. After the analysis was completed and the forms were collected, a brief meeting was held with the panelists to gather additional feedback, which was subsequently addressed in the results and discussion section.

##### Texture Profile Analyses

Texture profile analyses (TPA) were performed using a Brookfield CT3 Texture Analyzer (Brookfield Engineering Inc., Middleboro, MA, USA). TPA was carried out in accordance with the method of Bourne [27], with minor modifications. The TPA parameters assessed included hardness (N), adhesiveness (mJ), chewiness (N.mm), resilience, cohesiveness, and springiness (mm) [28]. For each gravlax fillet, 2 × 2 cm samples were excised from four different positions after equilibration to room temperature (24 °C), with a total of six replicates per group (*n* = 4). The TPA analysis was performed using a cylindrical probe with a diameter of 12.20 mm. The operational parameters were set as follows: triggering sensitivity of 0.05 N, probe speed of 2 mm/s, compression rate of 60%, recovery time of 2 s, and maximum load of 5 kg.

##### Mineral Substances and Heavy Metals Analyses

Mineral and heavy metal analyses were performed at the Sinop University Central Laboratory Facility (SÜBİTAM, Sinop, Türkiye). Analyses were carried out according to the Environmental Protection Agency (EPA) Method 200.3 protocols [29]. Each analysis was conducted in triplicate (*n* = 3). Briefly, approximately 1 g of meat samples was placed into the Teflon vessels and digested using a mixture of concentrated HNO_3_ (suprapur, 65%) and H_2_O_2_ (suprapur, 30%) at a ratio of 7:1. Digestion was conducted using a microwave digestion system (Milestone Corp., Shelton, CT, USA) following the temperature and pressure profile recommended by the Milestone SK-10 High Pressure Rotor (HPR-FO-67) application manual [30]. After adding the acid mixture, the Teflon vessels were sealed, heated gradually to 200 °C over a period of 15 min, and maintained at this temperature for an additional 15 min. Following the digestion process, the samples were transferred into 50 mL polypropylene Falcon tubes, diluted to 50 mL with ultra-pure water, and subsequently analyzed using an Agilent Technologies 7700x Inductively Coupled Plasma Mass Spectrometer (ICP-MS) (Agilent Technologies Inc., Santa Clara, CA, USA). Quality assurance and quality control (QA/QC) procedures included triplicate analyses and the use of certified reference material (CRM), Lobster TORT-2. Multi-element standard solutions, provided by Agilent (27-element mix: 8500-6940 2A and mercury standard 8500-6940 Hg), were used to prepare calibration curves. Analytical precision was maintained within ±5%. Additionally, a 1 ppm internal standard (Agilent 5188-6525, Agilent Technologies Inc., Santa Clara, CA, USA) was continuously analyzed alongside samples to monitor instrument stability. CRM recovery accuracy ranged between 95% and 100%. The limits of detection (LOD) and quantification (LOQ) for each element are presented under the calibration curves.

##### Statistical Analyses

All analytical data are presented as mean ± standard error. Following verification of homogeneity of variances, a one-way analysis of variance (ANOVA) was performed for each analysis at the *p* < 0.05 significance level using the Minitab 22.0 software package (Minitab Inc., State College, PA, USA). Chi-square tests were conducted using SPSS software (Version 27.0 for Windows, IBM Corp., Armonk, NY, USA) to assess data normality. In addition, multivariate analyses, including principal component analysis (PCA), partial least squares (PLS), and Pearson’s correlated polar heatmap with dendrogram, were performed, and all graphical visualizations were generated using OriginPro 2025 (OriginLab Corporation, Northampton, MA, USA).

## 3. Results and Discussion

### 3.1. Color

**Figure 4:**
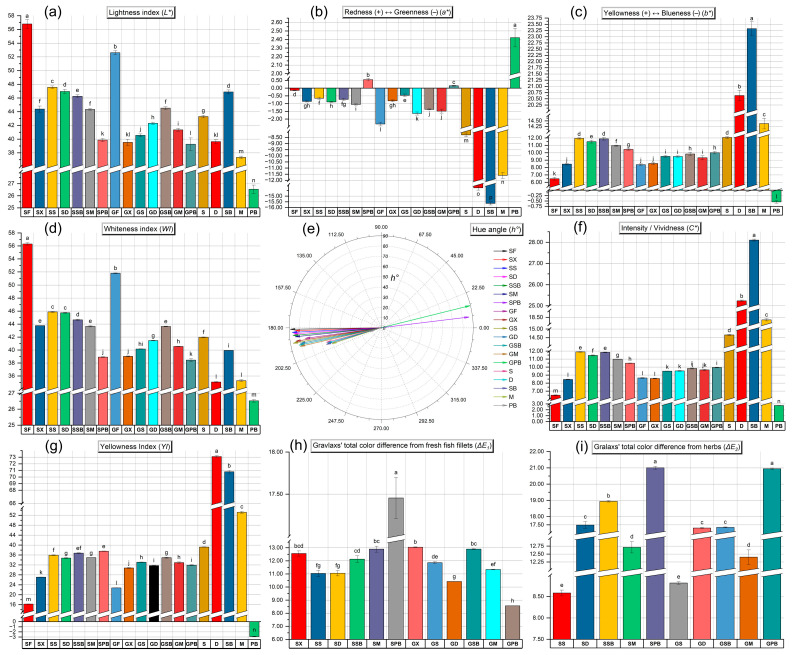
Results of color analyses. (**a**) L*; (**b**) a*; (**c**) b*; (**d**) *WI*; (**e**) *h°*; (**f**) C*; (**g**) *YI*; (**h**) *∆E*_1_; (**i**) *∆E*_2_. In each graph, labels on the bars with different letters are significantly different from each other (*p* < 0.05).

It is well established that food color significantly influences consumer perceptions of quality, especially in shaping their judgments of flavor, aroma, origin, naturalness, and ripeness [16,31]. In all gravlax samples produced using different herbs, significant color changes were observed compared to fresh fish fillets (*p* < 0.05) (Figure 4).

The L* values of all samples ranged from 26.58 ± 0.24 to 56.79 ± 0.14. The L* value in fish and their processed products is influenced by numerous factors such as muscle structure, chemical reactions, thickness, water content, pigment composition, and processing conditions [32]. The raw tub gurnard and garfish fillets (SF and GF) exhibited the highest L* values, indicating the brightest appearance among all groups. In contrast, the SPB and GPB groups displayed a significantly darker appearance (*p* < 0.05). The lower L* values observed in the groups produced with purple basil (PB) are attributable to the transfer of dark-colored pigments from this herb to the fillets (Figure 4a). In the SX and GX groups, where no herb material was used, the direct influence of salt, sugar, and other spices was evident. Additionally, it was found that these groups exhibited the lowest moisture content. Huang et al. [32] reported a strong positive correlation between water loss and the L* parameter in yellow croaker (*L. crocea*) fillets. Our findings also demonstrate a high degree of overlap between the moisture data and L* values (r = 0.88). Among the various herbs used, the highest L* values were recorded for SB, S, D, M, and PB, respectively. Erikson and Misimi [33] noted that increasing pH levels resulted in higher L* values in salmon fillets. In our study, correlation coefficients of 0.86 and 0.63 were observed between the pH levels detected in the herbs and the L* values of tub gurnard and garfish gravlax produced with these herbs, respectively. However, it should be noted that the notably high acidity of dill (D) compared to the other herbs exerts a diminishing effect on this correlation.

Positive a* values were observed exclusively in purple basil (PB) and in gravlax products containing purple basil (Figure 4b). Among these, the highest to lowest positive a* values were detected in the PB, SPB, and GPB groups, respectively (*p* < 0.05). In contrast, significantly negative a* values were found in all other herb groups except for purple basil (*p* < 0.05). This negative a* value is an expected outcome due to the high chlorophyll content in these herbs. However, in herbs rich in carotenoids, betalains, or anthocyanins, the a* value tends to shift in the positive direction [34,35]. Owing to its high anthocyanin content, the a* value for purple basil also shifted in the positive direction [36]. The most negative a* values detected among the herbs were found in the SB, D, M, and S groups, respectively (*p* < 0.05). The a* values of fresh tub gurnard and garfish fillets were determined to be −0.11 and −2.24, respectively. As observed in fresh fish fillets, substantial differences in a* values exist among fish species. While the addition of salt to tub gurnard fillets caused a significant decrease in a* value compared to the fresh fillet, the opposite effect was observed in garfish, where the a* value increased in the salted group compared to the fresh fillet. These findings can be attributed to the differences in muscle structure and the reactions with salt between darker, fattier fish and lighter, leaner fish species [37].

The b* value of sweet basil (SB) was found to be the highest among all groups (*p* < 0.05) (Figure 4c). This was followed by dill (D), mint (M), and sage (S), respectively. In contrast, the b* value of PB was positioned on the negative side (*p* < 0.05). The SX group represents the gravlax prepared without any herbs; therefore, only the effects of salt, sugar, and spices are expected to be observed in this group. Compared to fresh tub gurnard fillets, the b* values in the SX group increased, whereas no significant difference was found between the herb-free garfish group (GX) and the garfish fillets (GF) (*p* > 0.05). Indeed, the b* value of fresh garfish fillets was already higher than that of tub gurnard fillets (*p* < 0.05). While the b* values significantly increased in tub gurnard, which has a lighter muscle color, following the addition of salt, sugar, and herbs, the increase was minimal in garfish, which possesses a darker muscle color, even after the inclusion of all these ingredients. Interestingly, despite the negative b* value of PB, the GPB group was identified as one of the groups with the highest positive b* values among garfish gravlax samples. As expected, in the tub gurnard, the b* value of the group prepared with PB was found to be lower than that of other tub gurnard gravlax groups (*p* < 0.05).

The whiteness index (*WI*) plays a critical role in consumers’ perception of products as “fresh” [38]. In fact, the concept of *WI* facilitates our understanding of food quality by combining the L*, a*, and b* parameters into a single value through various mathematical formulas [16]. For this reason, *WI* is an especially important criterion for gourmet products such as seafood, where visual quality is paramount. In both fish species, the fresh fillets exhibited the highest *WI* scores; however, due to the lighter flesh color of the tub gurnard, its fillets had a significantly higher *WI* score compared to garfish fillets (*p* < 0.05). Among the herbs, *WI* scores were determined as follows: S > SB > M = D > PB (Figure 4d). In both fish species, the infusion of salt and sugar into the flesh resulted in a decrease in *WI* values, but the use of herbs led to an increase in *WI* values, particularly in tub gurnard gravlax. Gravlax produced with purple basil (PB) exhibited the lowest *WI* values, while the groups prepared with basil (SB) were identified as those with the highest *WI* scores in both fish species.

The yellowness index (*YI*) is commonly used to detect chemical deterioration, dehydration, light exposure, or excessive processing in foods; however, in the specific context of this study, the *YI* parameter is directly related to pigment transfer from the herbs, the presence of salt, sugar, and spices, as well as differences between fish species. Dill (D) exhibited by far the highest *YI* level, followed by SB (Figure 4g). In contrast, PB demonstrated a negative *YI* value. The effect observed in the gravlax groups was at a level similar to that of the *WI* parameter.

Total color differences (*ΔE*) can be calculated as the modulus of the distance vector between the initial color values and the actual color coordinates [16,39]. *ΔE*_1_ represents the color changes between fresh fish fillets and gravlax samples, while *ΔE*_2_ indicates the difference between the colors of the produced gravlax and those of the herbs. Analytically, differences in perceivable color are typically categorized as very distinct when *ΔE* exceeds 3, distinct when *ΔE* is between 1.5 and 3, and as a slight difference when *ΔE* is less than 1.5 [40]. The highest *ΔE*_1_ value was observed in the SPB group, whereas the lowest was found in the GPB group (Figure 4h). In other words, the GPB group remained closest to the color of fresh garfish flesh, with the combination of salt, sugar, and purple basil herb preserving its original color. Conversely, the same herb and processing conditions in the SPB group had the opposite effect, resulting in the highest color difference between tub gurnard flesh and this group. In terms of the difference between the herbs and gravlax samples, the highest *ΔE*_2_ value was detected in the groups prepared with PB (Figure 4i). These were followed by the groups containing SB and D, respectively.

**Figure 5:**
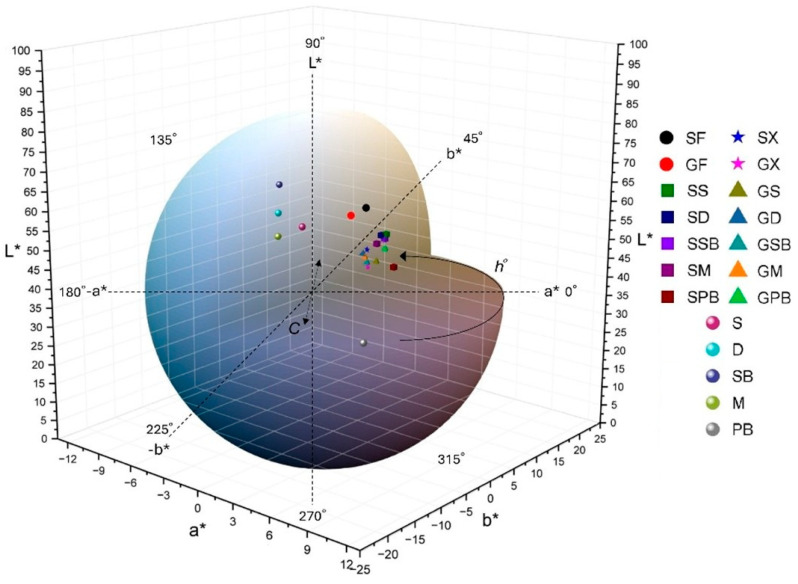
Distribution of 3D color parameters of gravlax groups, fillets, and herbs in *CIELAB* color space [14].

The hue angle (*h°*), regarded as the qualitative attribute of color, refers to the traditional way in which colors are described as reddish, greenish, etc., and is used to define the difference of a specific color from a gray color of the same lightness. This property is related to differences in absorbance at various wavelengths [16]. As the *h°* value increases, there is a decrease in yellowish tones and an increase in reddish-blue (purplish) tones; conversely, a negative *h°* indicates bluish-green tones [16]. The SPB and GPB groups exhibited positive *h°* values, whereas the other groups were positioned in the negative direction (Figure 4e). A remarkable observation here is that, while the groups produced with PB displayed positive *h°* values, the herb itself was positioned on the opposing side. The critical point in this context is the balance of hue difference; that is, the extent to which the color of the groups deviates from gray within their own color space, which gives rise to such findings. In representative color illustrations generated using computer-based *CIELAB* parameters (Figure 5), the differences in hue become more apparent.

**Figure 6:**
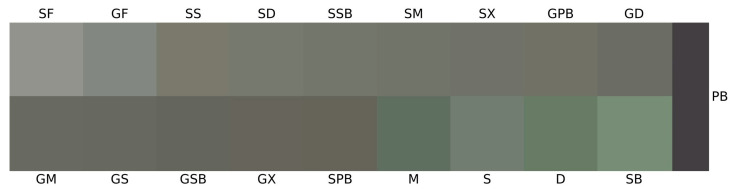
Computer-based color chart illustrations of gravlax groups and herbs.

The chroma (C*) value can be defined as the color saturation or intensity perceived by humans. Among the herbs, the highest and lowest C* values were observed in SB and PB, respectively (Figure 4f and Figure 5). It was also determined that the impact of these saturation levels in herbs was more pronounced in tub gurnard, whereas such an effect was not observed in garfish due to its darker flesh color (Figure 6).

### 3.2. pH, aw, and Salt

**Figure 7:**
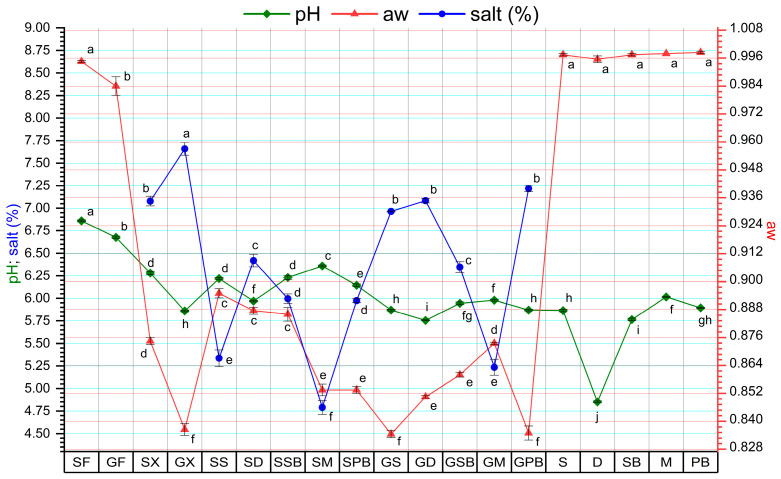
pH, aw, and salt (%) findings of gravlax groups and herbs. Each point in the same colored line, labeled with different letters, is significantly different from each other (*p* < 0.05).

The pH and aw values of fresh tub gurnard and garfish were determined to be 6.86 ± 0.02, 6.68 ± 0.03, 0.99 ± 0.00, and 0.98 ± 0.00, respectively (*p* < 0.05). Following the production of gravlax from fresh fish fillets, a decrease was observed in the pH and aw values of all gravlax groups (*p* < 0.05) (Figure 7). In terms of fish species, a high level of correlation was found between the changes in pH and aw in the garfish group (r = 0.87), while a moderate correlation was observed in the tub gurnard group (r = 0.75). Tönißen et al. [41] have reported that pH is influenced by several factors, including size, spawning season, freshness, storage conditions, and intra-species genetic factors. Altan et al. [2] reported that, in gravlax produced from bonito (*S. sarda*) with dill (*A. graveolens*) and garden cress (*L. sativum*), the lowest pH was found in the fresh fish meat, while the highest pH was determined in the garden cress group. In contrast to these findings, our study revealed that the groups with the highest pH values were those consisting of fresh fish fillets (*p* < 0.05), while the gravlax group with the lowest pH was the GD group (*p* < 0.05). Furthermore, among the herbs used, dill was found to have the lowest pH value (*p* < 0.05). Similar to previous studies, it was also determined that the groups containing dill exhibited the lowest pH values.

When examining the salt (%) content of the groups, it was observed that garfish samples contained higher levels of salt (Figure 7). It is well known that tissue thickness plays a significant role in the penetration of salt into the flesh. Similarly, due to osmoregulation, gravlax produced from garfish exhibited lower aw and moisture content compared to tub gurnard gravlax. The highest salt contents were determined in gravlax samples produced without the addition of any herbs (GX and SX). In these groups without herbs, since there is no water influx into the flesh or osmoregulatory interaction between the flesh and herbs, higher salt absorption is expected. Among the groups containing herbs, mint gravlax (GM and SM) was found to have the lowest salt content (*p* < 0.05). In both fish species, gravlax groups containing dill consistently had the highest salt content. In gravlax produced with sage (S), its use was found to increase salt absorption in garfish fillets, while leading to lower salt uptake in tub gurnard fillets. Such differences in salt content between groups may influence sensory preferences.

### 3.3. Nutritional Composition

The total protein, lipid, moisture, ash, carbohydrate, and energy contents of fresh tub gurnard and garfish fillets were determined to be 16.13 ± 0.06%, 2.18 ± 0.02%, 79.06 ± 0.11%, 2.49 ± 0.05%, 0.11 ± 0.01%, and 84.93 ± 0.26 kcal and 17.78 ± 0.05%, 3.11 ± 0.04%, 76.20 ± 0.08%, 2.65 ± 0.04%, 0.21 ± 0.03%, and 100.22 ± 0.37 kcal, respectively. According to data from the Turkish National Food Composition Database [42], the protein, lipid, moisture, ash, carbohydrate, and energy values of garfish available throughout the year were reported as 20.24%, 4.93%, 73.10%, 1.44%, 0.01%, and 125 kcal, respectively. Duyar & Özdemir [43] reported the protein, lipid, moisture, ash, carbohydrate, and energy contents of tub gurnard (n = 30) as 19.64%, 3.23%, 75.69%, 1.54%, 0.36%, and 143.06 kcal, respectively. As can be seen, the nutritional composition of the tub gurnard and garfish samples used in our study showed some variations compared to previous literature. It is well known that numerous factors, including environmental conditions, spawning period, size and weight differences, and sex, can affect the nutritional composition of fish.

**Figure 8:**
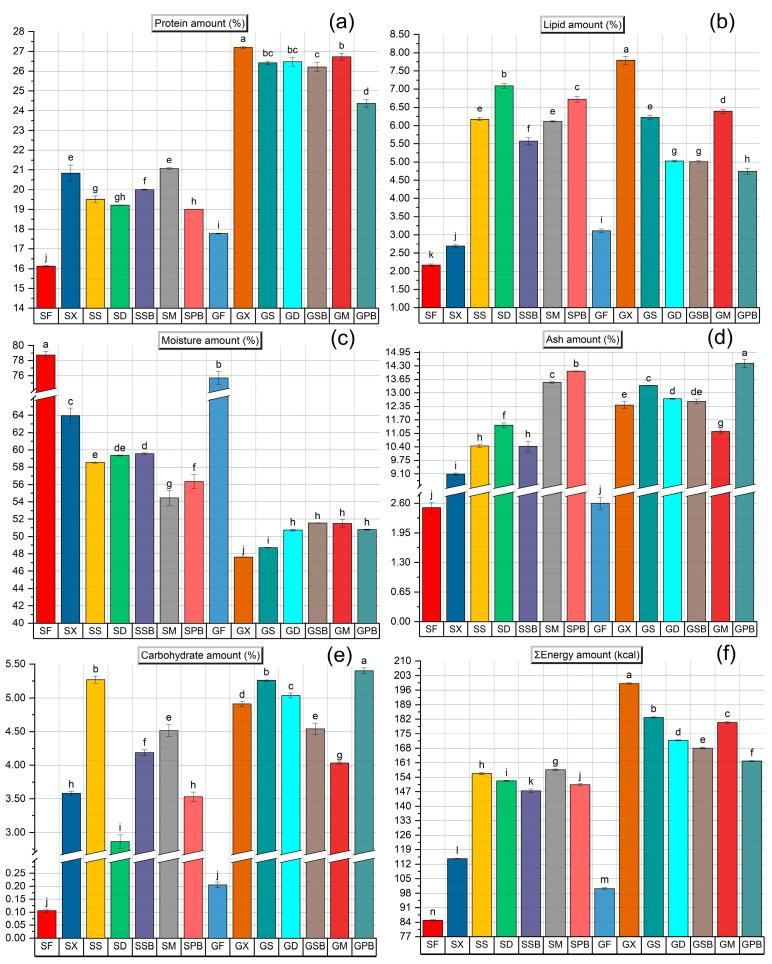
Nutritional composition of gravlax groups and fresh fish fillets. (**a**) Protein amount (%); (**b**) lipid amount (%); (**c**) moisture amount (%); (**d**) ash amount (%); (**e**) carbohydrate amount (%); (**f**) ∑energy amount (kcal). In each graph, labels on the bars with different letters are significantly different from each other (*p* < 0.05).

During gravlax production, due to the influx of salt and sugar into the fish fillets, a decrease in moisture content was observed in all gravlax groups (*p* < 0.05) (Figure 8). Following osmoregulation, the reduction in water content resulted in a proportional increase in protein, lipid, ash, carbohydrate, and energy levels in all gravlax groups (due to moisture loss and sugar addition) (*p* < 0.05).

It was determined that the protein content of the SPB and GPB groups produced with PB was significantly lower (*p* < 0.05) (Figure 8a). Additionally, it was observed that the ash content of these groups produced with PB was at a higher level (Figure 8d). Commonly, among the samples, the gravlax produced without any herb addition (X) exhibited the highest protein content, while the gravlax prepared with mint (M) yielded results closely comparable to those without herb additives. This finding suggests that mint may be effective in preventing protein loss. When examined by fish species, the protein content of gravlax produced from tub gurnard was found to range between approximately 19% and 21%, whereas gravlax produced from garfish ranged between 24% and 27%. The World Health Organization (WHO) recommends a daily protein intake, known as the recommended daily allowance (RDA), of 0.80 g per kg of body weight for adults [44]. Accordingly, a healthy adult weighing 75 kg should consume approximately 60 g of protein per day. A single portion of gravlax (57 g) [6] provides between 20% (when produced from gurnard) and 25% (when produced from garfish) of the recommended daily protein requirement. This clearly highlights the nutritional value of gravlax as a protein-rich seafood option, offering a significant contribution to meeting daily protein needs within a balanced diet.

The changes in ash content and the corresponding salt variations exhibited a strong positive correlation in tub gurnard (r = 0.91), while a moderate positive correlation was observed in garfish (r = 0.76). The relatively lower correlation in garfish can be attributed to the inherently higher and more dominant mineral content of this species. Indeed, it is evident from the mineral content analysis that both the transfer of minerals from herb additives and the pronounced interspecies differences play a significant role in the final mineral composition of the fish products.

It was determined that tub gurnard fillets contained a higher moisture content compared to garfish fillets (Figure 8c). Such species-related differences are to be expected among various types of fish. While the moisture variation in gravlax produced from garfish ranged between 48% and 52%, this range was found to be approximately 54% to 64% in gravlax made from tub gurnard. As can be seen, the gravlax produced from the tub gurnard, which has a thicker muscle structure, exhibited a broader range of moisture content. The penetration of salt, sugar, and spices may increase or decrease to some extent, depending on fillet thickness. Fillet thickness directly influences many physical and organoleptic parameters in gravlax production [1].

Although the initial lipid content of garfish fillets was significantly higher than that of tub gurnard fillets (*p* < 0.05), after post-gravlax production, the lipid contents of the fillets became more comparable between the two species (Figure 8b). Except for the SX (2.54 ± 0.13%) group, the lipid content of the gravlax samples ranged from approximately 5% to 7.80%. It can be stated that while the increase in lipid content was very limited in tub gurnard gravlax without herb additives, the gravlax produced from garfish without herb additives exhibited the highest lipid content among all gravlax samples. This phenomenon is thought to be related to other nutritional components, as the GX group, which had the highest lipid content (7.79 ± 0.12%), also demonstrated the lowest moisture and the highest protein content (*p* < 0.05). Considering that this group was produced as gravlax without herb additives, it is particularly important for reference purposes.

The carbohydrate content of all gravlax groups increased compared to fresh fish fillets (*p* < 0.05) (Figure 8e). This increase in carbohydrate levels is an expected outcome, particularly due to the addition of sugar to the fillets. When the energy values are examined, it can be observed that, overall, gravlax produced from garfish possessed a higher energy content than those produced from tub gurnard (*p* < 0.05). The energy value of the SX group (114.68 ± 0.23 kcal) was determined to be lower, which can be attributed to its lower lipid content relative to other groups (Figure 8f). It should be noted that a multitude of parameters influence energy values.

### 3.4. Amino Acids

**Figure 9:**
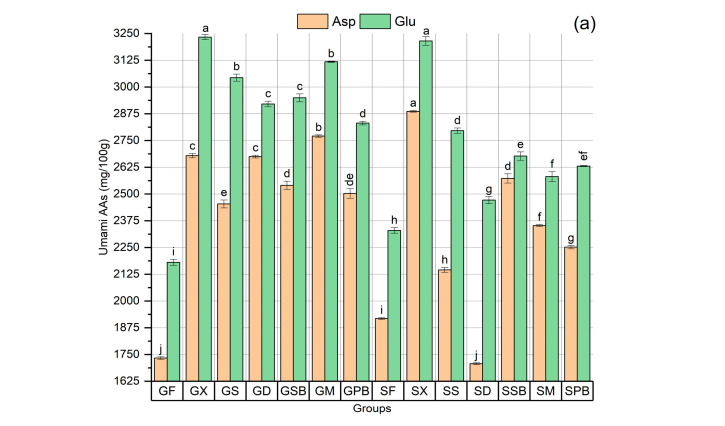

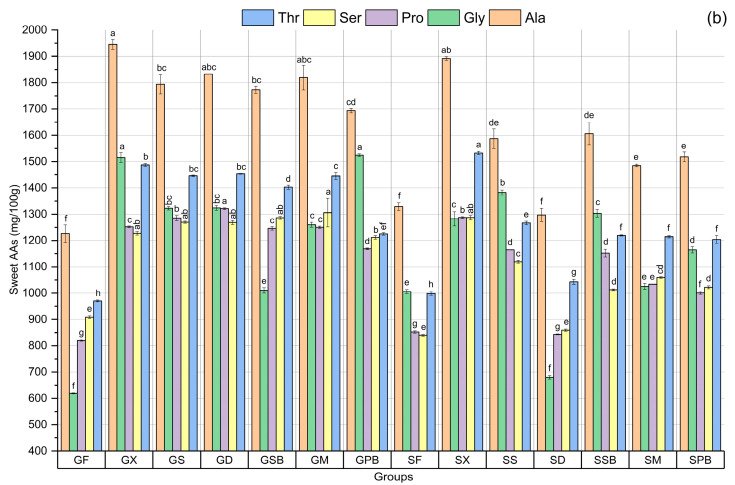

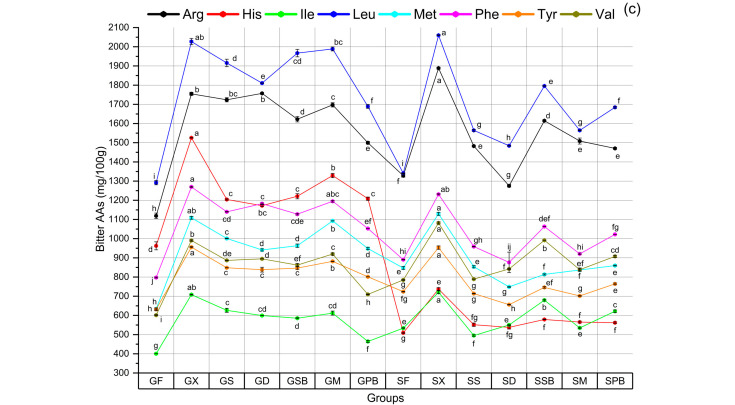

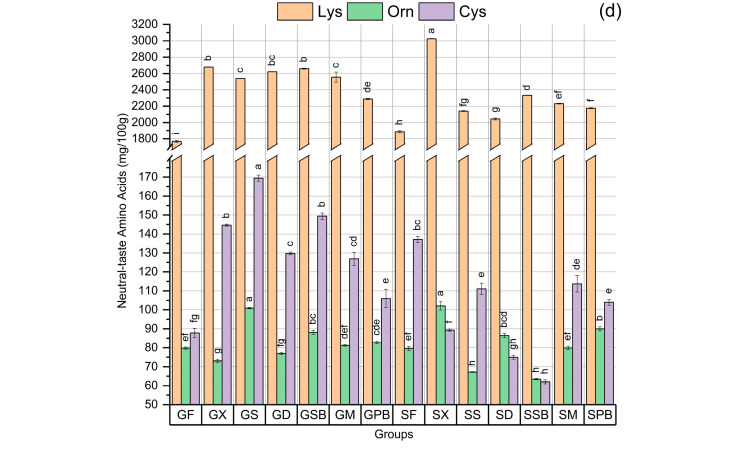

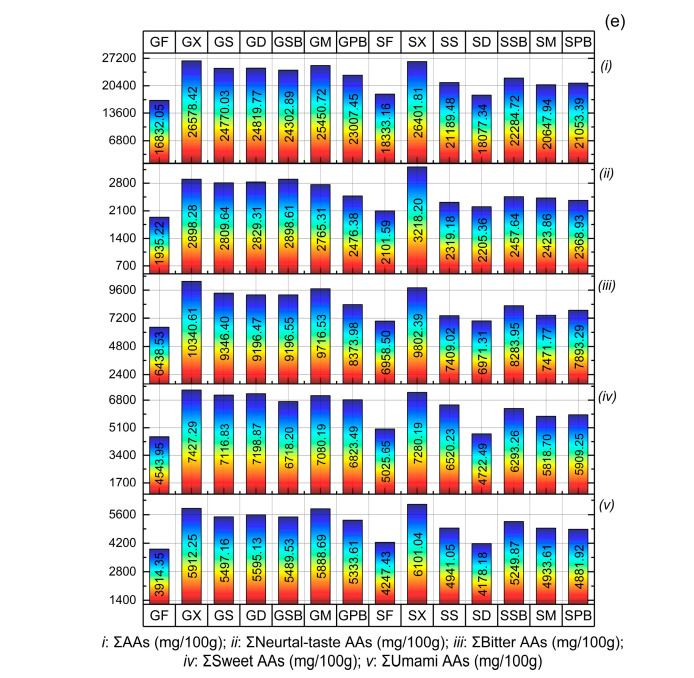
Amino acids analysis results: (**a**) umami amino acids profile; (**b**) sweet amino acids profile; (**c**) bitter amino acids profile; (**d**) neutral-taste amino acids profile; (**e**) total (Σ) amino acid contents according to taste classification. In each graph, labels on the bars or line points with different letters are significantly different from each other (*p* < 0.05).

Amino acids were categorized according to the taste classification proposed by Kaneko et al. [45]. In terms of total amino acid content, the SX and GX groups were identified as those containing the highest amounts of amino acids (Figure 9e). Notably, both of these groups represent gravlax produced without herbs, which may indicate increased levels of free amino acids, typically resulting from enhanced proteolytic degradation. Several researchers have reported that both the total amino acid content and the levels of amino acids associated with umami and sweet taste profiles, such as Glu, Leu, Ala, Gly, and Asp, increase in low-salt fermented fish products [46,47]. The observation that these two groups generally possess higher levels of total umami and sweet amino acids, as well as particularly elevated amounts of Leu, Gly, and Ala, further supports this finding (Figure 9a,b,e). The generally lower amino acid content detected in gravlax produced with herbs may suggest that herbs slow down proteolytic degradation. Varied effects of herbs on proteolytic activity have been observed. Among the gravlax samples containing herbs, the use of mint (M) in garfish gravlax resulted in a slightly higher amino acid content, while sweet basil (SB) increased amino acid levels in tub gurnard gravlax. Conversely, the inclusion of dill (D) led to a decrease in the total amino acid content in tub gurnard gravlax, yet this decline was not observed in gravlax produced from garfish with dill (D). Similarly, purple basil (PB) caused a slight decrease in the total amino acid content of garfish gravlax, while it did not result in a significant change in tub gurnard gravlax. Additionally, among the gravlax samples produced from garfish, the GPB group had the lowest protein content (*p* < 0.05), whereas the SD group had the second lowest protein content among the tub gurnard gravlax samples (Figure 8a).

Glutamic acid and aspartic acid are amino acids responsible for the formation of the umami taste [46]. The overall ranking of total umami content among the garfish groups was determined as GX > GM > GSB = GS > GD > GPB > GF, while among the tub gurnard groups, it was observed as SX > SSB > SM = SS > SPB > SD = SF. As can be seen, in both fish species, the highest umami values were detected in the groups without herbs (X), whereas the lowest values were found in the groups with fresh fillets combined with dill (D) and purple basil (PB). Groups containing mint (M) and basil (SB) also exhibited relatively high levels of umami. It is evident that herbs have a significant impact on umami content (Figure 9a). Notably, when the umami amino acids identified in the gravlax samples produced from each fish species were correlated with the responses to the “umami feeling” parameter from sensory analysis, the correlation coefficients (r) were found to be 0.90 and 0.92 for garfish and tub gurnard with herbs, respectively.

Among garfish, the ranking of amino acids responsible for sweet taste was determined as GX > GS = GD > GM > GPB = GSB > GF, while in tub gurnard, the sequence was identified as SX > SS > SSB > SM = SPB > SF > SD. The groups without herbs exhibited the highest levels of sweet-tasting amino acids, whereas, among the herb-containing groups, those prepared with sage (S) consistently displayed higher sweet amino acid levels (Figure 9b,e). When the scores for the “sweetness-liking” parameter from sensory analyses were correlated with the levels of sweet amino acids in the gravlax groups, the correlation coefficients (r) for garfish and tub gurnard with herbs were found to be 0.88 and 0.83, respectively. These results indicate a strong association between the sensory analysis parameters and the groups of amino acids responsible for specific tastes.

The ranking of bitter-tasting amino acids in tub gurnard gravlax was determined as SX > SSB > SPB > SM = SS > SD = SF, whereas in garfish gravlax, the order was GX > GM = GS > GSB = GD > GPB > GF (Figure 9c). The groups without herbs (X) exhibited the highest bitter amino acid contents, while the fresh fillet (F) and SD groups were identified as those with the lowest levels of bitter amino acids. Among the sensory parameters, it was observed that only the “sourness liking” parameter demonstrated a moderate correlation with the levels of bitter amino acids. The correlation coefficients (r) for garfish and tub gurnard in relation to herbs were 0.71 and 0.82, respectively.

Amino acids responsible for natural taste were generally present in similar proportions across groups. In the garfish and tub gurnard groups, the rankings were GX = GSB = GD = GS = GM > GPB > GF and SX > SSB = SM = SPB = SS = SD > SF, respectively. In both fish species, natural-tasting amino acids were found at the lowest levels in fresh fillet (F) samples (Figure 9d).

The findings regarding amino acids indicate that herbs exert different effects on gravlax produced from different fish species; however, these differences were found to be in moderate to high agreement with the results from sensory analyses.

#### 3.4.1. Principal Component Analysis of Amino Acid Profiles

**Figure 10:**
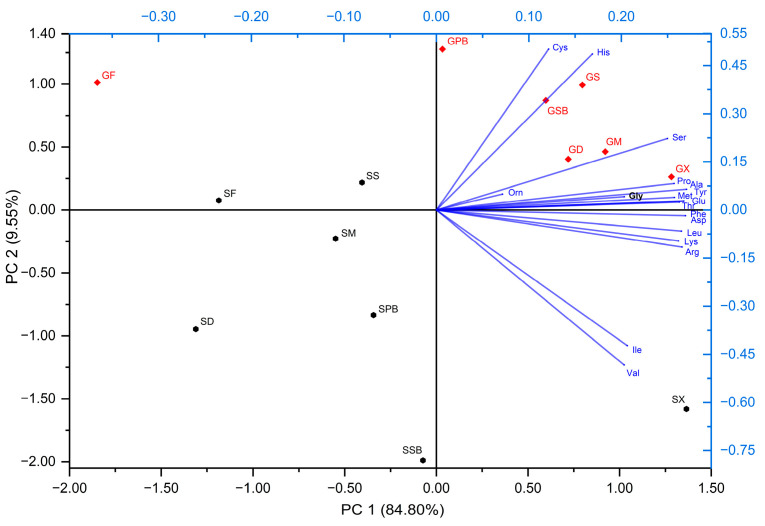
PCA analysis graph of amino acids. The lines (loading plots) and text in blue represent amino acids. The groups in red represent garfish fillets and gravlax produced from garfish, while the groups in black represent tub gurnard fillets and gravlax produced from tub gurnard.

Principal component analysis (PCA) effectively revealed distinctive patterns in amino acid composition, facilitating clear differentiation among sample groups. The first two principal components captured a substantial portion of the total variance, accounting for 94.35% collectively, with PC1 explaining 84.80% and PC2 contributing 9.55% (Figure 10).

The loading matrix indicates that PC1 displays positive loadings across all amino acids, ranging from 0.121 for Cys to 0.270 for Ala (Table 2). This consistent positive loading pattern suggests that PC1 represents a general gradient related to overall amino acid concentration or total amino acid content. Among amino acids, Ala (0.270), Phe (0.269), Glu (0.267), Arg (0.266), and Leu (0.265) contribute most significantly to PC1, emphasizing their pivotal role in defining the primary axis of variation. On the other hand, PC2 exhibits a clear bipolar structure, distinctly segregating amino acids into two separate groups. Amino acids such as Cys (0.501), His (0.486), and Ser (0.223), along with others showing moderate positive loadings, form one distinct group (Table 2). Conversely, amino acids such as Val (−0.483), Ile (−0.422), Arg (−0.115), Lys (−0.096), and Asp (−0.067) exhibit negative loadings. This bipolar distribution suggests that PC2 reflects specific metabolic or functional differences between these amino acid groups, potentially indicating variations in biochemical pathways or protein functionalities. Overall, the interpretation of principal component loadings suggests that PC1 primarily captures variations in amino acid profiles arising from interspecies differences among fish, thereby allowing more precise differentiation between species. In contrast, the patterns observed along PC2 indicate that the grouping or polarization of specific amino acids is predominantly associated with variability introduced by different herb types rather than inherent interspecies differences. Thus, species-driven variations are mainly represented by PC1, whereas herb-derived influences on amino acid composition become more evident along the PC2 axis.

#### 3.4.2. Individual Group Distribution and Separation of Amino Acids

Examination of the biplot visualization revealed a distinct spatial separation among sample groups along both principal component axes (Figure 10). Samples marked in red (garfish groups) predominantly clustered within the positive regions of both PC1 and PC2, whereas samples marked in black (tub gurnard groups) occupied the negative region of PC1. This clear separation strongly indicates fundamental differences in amino acid profiles between these two sample categories. Specifically, the red-marked groups are characterized by a higher overall amino acid content with elevated levels of Cys, His, and Ser, whereas the black-marked groups exhibit lower total amino acid levels but relatively higher concentrations of Val and Ile.

#### 3.4.3. Correlation Between Identified Principal Components and Factors of Amino Acids

**Figure 11:**
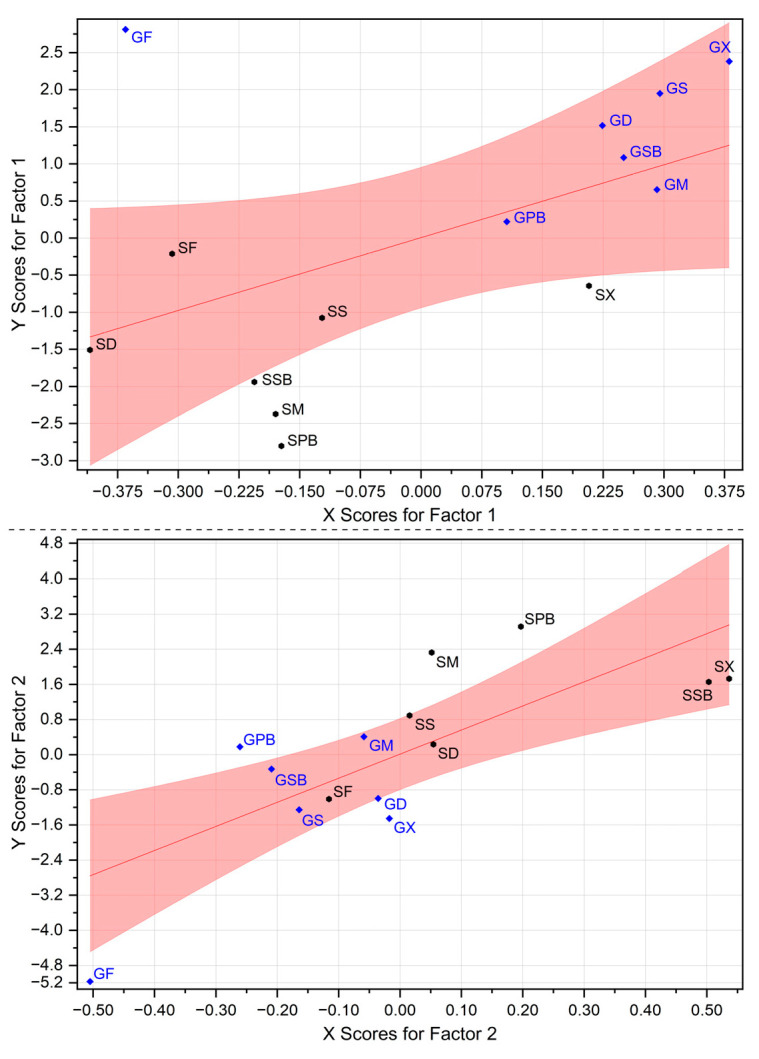
Correlation between identified PC components and factors of amino acids. The groups in blue represent garfish fillets and gravlax produced from garfish, while the groups in black represent tub gurnard fillets and gravlax produced from tub gurnard.

In this analysis, the statistical relationships between amino acid factor analysis coefficients and group positions in PC space were elucidated. The pink confidence band indicates a significant linear relationship between amino acid profiles and group positioning (Figure 11). The proximity of groups to the red regression line within the confidence band indicates stronger relationships. In Factor 1 analysis, samples marked in blue (garfish groups) clustered predominantly in the upper right quadrant of the plot, displaying high positive X-scores (ranging from 0.2 to 0.4) and elevated Y-scores (ranging from 0.5 to 2.5). Conversely, samples marked in black (tub gurnard groups) exhibited negative X-scores (ranging from −0.4 to −0.1) and lower or negative Y-scores (ranging from −2.0 to 0.5). This distribution clearly demonstrates a strong positive correlation between Factor 1 and amino acid composition. Factor 2, however, exhibits a more complex pattern of distribution. Within this factor, SPB and SM samples display the highest Y-scores (2.5–3.0), while the GF sample shows the lowest Y-score (−5.2) (Figure 11). Considering X-scores, SX and SSB samples are positioned at the positive extreme (0.4–0.5), whereas GF occupies the negative extreme (−0.5). This distribution suggests Factor 2 identifies specific variations in amino acid profiles, likely reflecting distinct herb-derived chemical reaction processes associated with PC2.

### 3.5. Sensorial Analyses

Prior to tasting the gravlax samples, participants were asked to consume a few small leaf pieces from each herb separately and provide detailed feedback about these herbs (Table S1). Following this initial herb tasting and evaluation, participants were requested to wait at least 15 min before proceeding to taste the gravlax samples. After completing the tasting session, participants filled out a detailed survey form (Table S2).

**Figure 12:**
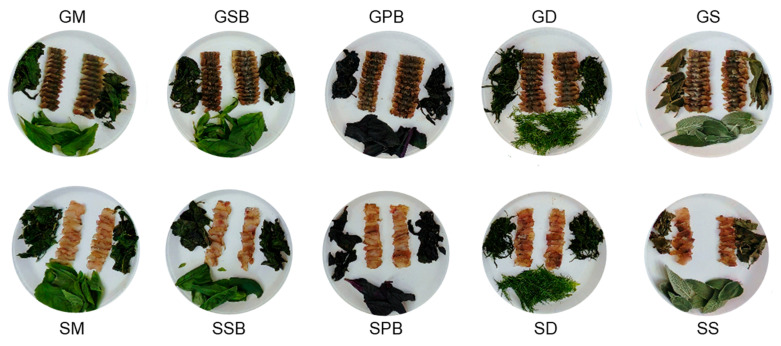
Sliced tub gurnard and garfish gravlax samples, and the herbs prepared for sensory analysis. For the assessment of visual appeal, both the wilted herbs present within the gravlax and their fresh counterparts were placed on the plates.

On the plates, both fresh herbs and herbs that had wilted as a result of the gravlax maturation process were presented together with the sliced gravlax (Figure 12). This presentation was intended to provide panelists with a more comprehensive understanding of the actual appearance and condition of the gravlax samples. Furthermore, panelists were also given the opportunity to observe the whole, unsliced gravlax fillets.

**Figure 13:**
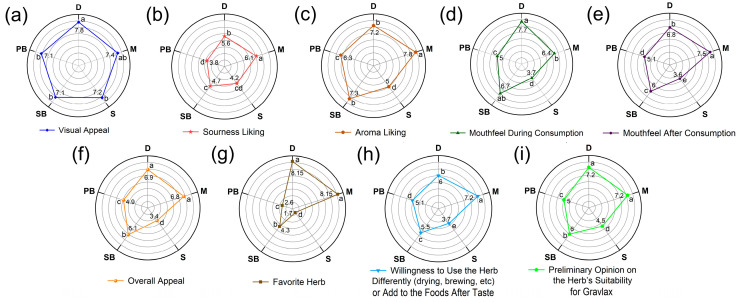
Sensory results of fresh herbs. (**a**) Visual appeal; (**b**) sourness liking; (**c**) aroma liking; (**d**) mouthfeel during consumption; (**e**) mouthfeel after consumption; (**f**) overall appeal; (**g**) favorite herb; (**h**) willingness to use the herb differently or add to the foods after taste; (**i**) preliminary opinion on the herb’s suitability for gravlax. In each radar graph, labels on the line points with different letters are significantly different from each other (*p* < 0.05).

Based on the initial sensory evaluation results (Figure 13), participants assigned the highest scores to mint (M) and dill (D) in response to the pre-tasting bias question regarding “preliminary opinion on the herb’s suitability for gravlax” (Figure 13i), even before tasting the products (*p* < 0.05). These herbs were followed by basil (SB), while sage (S) received the lowest scores (*p* < 0.05). Regarding participants’ openness to using these herbs in various forms (drying, brewing, etc.), mint (M) received the highest scores (Figure 13h) and sage (S) the lowest (*p* < 0.05). Dill (D) and mint (M) were rated highest for sensations both during and after consumption (Figure 13d,e), while sage (S) consistently scored lowest in these categories (*p* < 0.05). Responses to the questions regarding “Overall appeal” (Figure 13f) and “Favorite herb” (Figure 13g) were identical, with preferences ranked as D = M > SB > PB > S (*p* < 0.05). Mint (M) exhibited the most “sourness-liking” scores (Figure 13b), while sage (S) and purple basil (PB) presented the least sourness-liking scores. For “Aroma liking,” mint (M) achieved the highest score, followed by sweet basil (SB) and dill (D), whereas sage (S) received the lowest ratings for aroma liking (Figure 13c). All herbs received similarly high scores for visual appeal; however, dill (D) and mint (M) were marginally preferred visually, although this difference was not statistically significant (*p* > 0.05) (Figure 13a). Based on the question, “Willingness to use the herb differently or consider using the herb in alternative ways (drying, brewing, etc.)?”, mint (M) received the highest scores, while sage (S) received the lowest (*p* < 0.05). Regarding the “mouthfeel during” and “after consumption”, dill (D) and mint (M) achieved the highest ratings, whereas sage (S) obtained the lowest scores (*p* < 0.05). Responses to the “Overall appeal” and “Favourite herb” questions were identical, with preference rankings established as D = M > SB > PB > S (*p* < 0.05). Visually, all herbs were similarly highly rated; however, dill (D) and mint (M) were slightly preferred, although this difference was not significant (*p* > 0.05).

**Figure 14:**
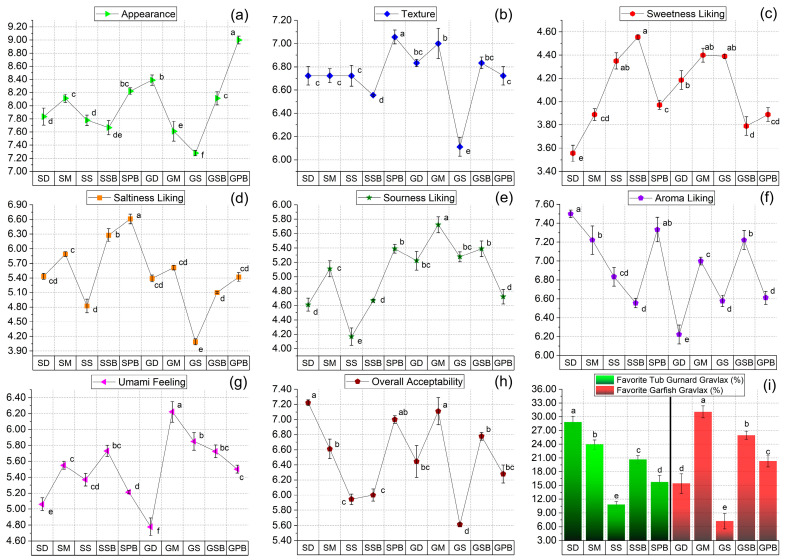
Sensory results of gravlax groups. (**a**) Appearance; (**b**) texture; (**c**) sweetness liking; (**d**) saltiness liking; (**e**) sourness liking; (**f**) aroma liking; (**g**) umami feeling; (**h**) overall acceptability; (**i**) favorite gravlax of garfish and tub gurnard. In each graph, labels on the line points or bar tags with different letters are significantly different from each other (*p* < 0.05).

Following the evaluation of herbs, participants proceeded to taste the gravlax samples. In terms of the “Appearance” parameter, GD and GPB received the highest scores, whereas the sage-infused garfish group (GS) was rated the lowest visually (Figure 14a). Regarding texture, the most favored groups were identified in the following order: SPB (7.06 ± 0.17), GM (7.02 ± 0.71), GD (6.84 ± 0.54), and GSB (6.80 ± 0.46) (Figure 14b). Texture scores for the groups SD (6.72 ± 0.25), SM (6.72 ± 0.13), SS (6.72 ± 0.31), and GPB (6.72 ± 0.18) were found to be similar (*p* > 0.05). Additionally, some panelists (*n* = 11) noted that the GS group was softer and less pleasant to chew compared to others, awarding it the lowest average score (6.11 ± 0.20) among all groups (*p* < 0.05).

For “Sweetness liking,” the highest scores were assigned to groups SSB, SS, GM, and GS, while lower levels of sweetness liking were perceived in SD, SM, GSB, and GPB groups (Figure 14c). In terms of “Saltiness liking,” all tub gurnard groups, except the sage-infused garfish group (GS), generally exhibited well-balanced saltiness (Figure 14d). Additionally, some panelists (*n* = 5) suggested in their notes that the combination of sage and salt enhanced each other’s effects, overpowering the fish flavor, and thus recommended lower salt levels when using sage specifically. Among tub gurnard samples, groups SSB and SPB had the highest saltiness liking scores, whereas garfish gravlax produced with the same herbs received slightly lower ratings. Panelists reported during a follow-up discussion that the salt balance in gurnard gravlax harmonized better with herb aromas. However, due to the darker and thinner texture of garfish meat, the saltiness was more pronounced, which affected the balanced taste typically experienced with gurnard gravlax. Regarding the “Sourness liking” parameter (Figure 14e), panelists generally assigned scores inversely proportional to their “Saltiness liking” ratings for garfish gravlax. They indicated that the pronounced saltiness in garfish positively influenced their perception of sourness, achieving a balanced taste. In terms of “Umami feeling,” dill (D) gravlax received the lowest scores across both fish species. Among the tub gurnard gravlax groups, the sweet basil-infused (SB) group provided the highest umami feeling (Figure 14g). Mint-infused (M) gravlax consistently received the highest umami ratings for both fish species, aligning closely with the “umami amino acids” identified in amino acid analyses (Figure 9a and Figure 14g). Panelists were asked to describe the sensations during the duration aroma was perceived in the mouth (Figure 14f) (“Aroma liking”). For tub gurnard, a mild-flavored white fish, dill (SD) was noted for a lingering pleasant sensation, whereas the same herb’s aroma presence and persistence in garfish (GD) were negligible and inadequate. Regarding “Overall acceptability,” the highest ratings were given to groups SD, GM, and SPB, while GS received the lowest (Figure 14h). When panelists were asked to select their top three gravlax types in the “Favorite gravlax (%)” category, the dill-infused tub gurnard group (SD) and mint-infused garfish group (GM) emerged as favorites, while sage-infused groups (SS and GS) were the least preferred (Figure 14i).

### 3.6. Texture Profile Analysis (TPA)

**Figure 15:**
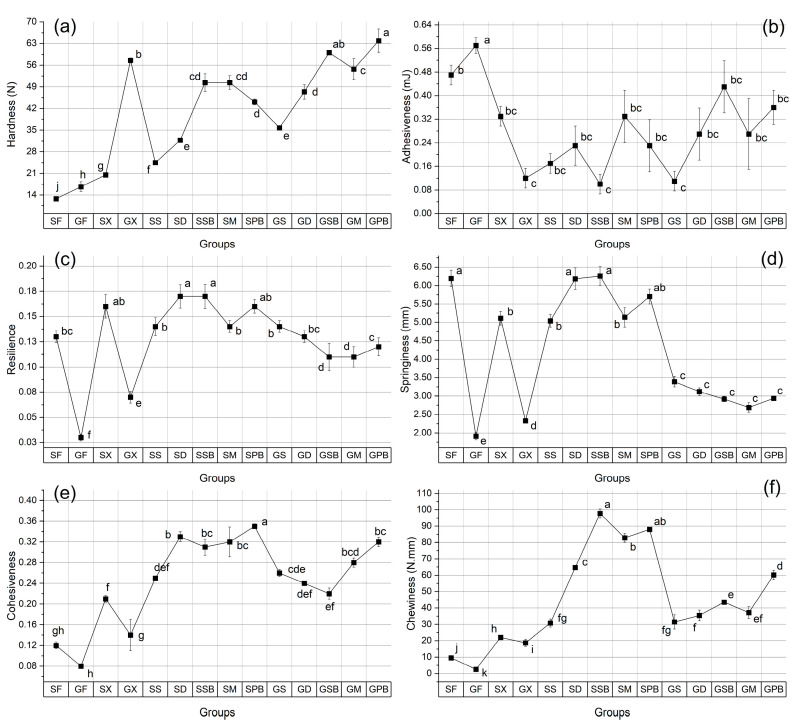
TPA results of fish fillets and gravlax groups: (**a**) hardness (N); (**b**) adhesiveness (mJ); (**c**) resilience; (**d**) springiness (mm); (**e**) cohesiveness; (**f**) chewiness (N.mm). In each graph, labels on the line points with different letters are significantly different from each other (*p* < 0.05).

The hardness (N) values for all groups increased compared to the fresh fish fillets, a result expected due to the moisture loss induced by the penetration of salt and sugar into the tissues (Figure 15a). Contrary to our hardness findings, Altan et al. [2] reported a significant decrease (*p* < 0.05) in hardness values of gravlax produced from bonito, likely attributable to the inherent properties of bonito itself, which is more susceptible to deformation compared to the fish species used in the present study. The highest hardness was recorded in the GPB group, while the lowest was observed in the SX group (produced without herbs) (*p* < 0.05). Apart from fresh fillets, GX exhibited the lowest moisture content, whereas SX showed the highest moisture content, explaining the observed differences in hardness between these groups. Examining only the herb-infused gravlax samples, the GS group had the second-lowest moisture content after the GX group, yet demonstrated an apparent softening effect of sage. The mint-infused groups exhibited a relatively uniform increase in hardness compared to fresh fillets. Sweet basil (SB) was consistently found to exert the most substantial hardness-increasing effect across both fish species.

**Figure 16:**
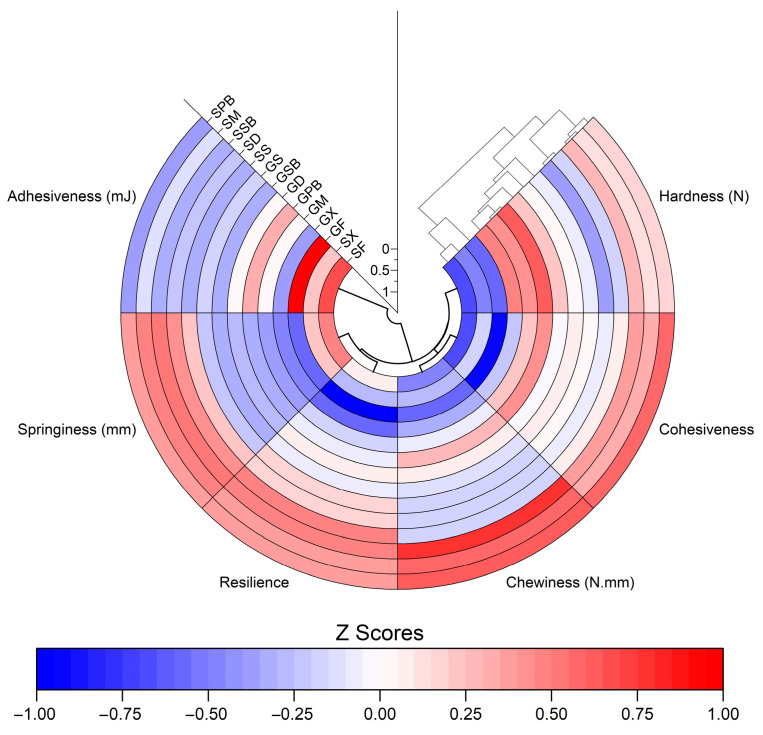
Polar heat map with dendrogram of gravlax groups.

The highest adhesiveness values were observed in fresh fish fillets, with a slight decrease observed across all other groups (Figure 15b). Due to their thinner texture and inherently higher hardness, garfish fillets exhibited significantly lower elasticity values compared to gurnard fillets, as expected (*p* < 0.05). Garfish gravlax, both with and without herbs, showed increased springiness values relative to fresh garfish fillets (*p* < 0.05). In contrast, the springiness values of gurnard gravlax remained broadly similar to those of fresh gurnard fillets (Figure 15d). Additionally, elasticity values among garfish gravlax infused with herbs were not significantly different from one another (*p* > 0.05). However, the springiness, resilience, and cohesiveness parameters (Figure 15c–e) were found to exhibit a very high correlation, with nearly identical trends across groups. The pairwise correlation coefficients (r) between springiness, resilience, and cohesiveness were determined to be 0.94, 0.91, and 0.95, respectively. Moreover, these relationships can also be clearly observed in the polarized heatmap, which was generated by standardizing the data using Z-scores (Figure 16). The highest adhesiveness parameters were determined in the groups of fresh fillets (Figure 15b). Among the garfish gravlax samples, the GPB group demonstrated the highest chewiness value, whereas the highest chewiness in gurnard gravlax samples was observed in the SSB group (Figure 15f). In general, gravlax samples prepared with sweet basil and purple basil consistently exhibited high chewiness, whereas samples prepared with sage showed the lowest chewiness values for both garfish and gurnard gravlax.

### 3.7. Mineral Substances and Heavy Metals

Among the major elements, the lowest sodium (Na) content was observed in the SF and GF groups (*p* < 0.05), as expected, since these values reflect the naturally occurring Na content in fresh fish meat (Table 3). After salt infusion, Na levels increased significantly. Gravlax fillets produced without herbs (GX and SX) had the highest Na contents, while herb-infused groups GD, GPB, GS, SSB, and SD exhibited relatively higher Na contents compared to other herb-infused groups. The GM and SM groups displayed the lowest Na contents. These findings are broadly similar to salt content analysis (Figure 7). The correlation between salt and Na content was analyzed separately for each fish species, with correlation coefficients (r) of 0.91 for garfish and 0.87 for tub gurnard. Erkan et al. [48] reported that the mineral content of fish and seafood products can vary considerably depending on both species and processing conditions and that major minerals, in particular, may undergo significant changes as a result of salting. In terms of potassium (K) and phosphorus (P) changes (Table 3), it was found notable that fresh fillets showed the highest levels. Moisture loss due to salting caused proportional weight changes, thereby increasing the concentrations of nearly all analyzed components. Gravlax produced without herbs also showed similar reductions in K and P, indicating that mineral losses occurred independently of the presence of herbs. This decrease in gravlax fillets is likely due to the leaching of K and P minerals, along with water, from the fish tissue. In terms of calcium (Ca) levels, the sweet basil (SB) groups had the highest values (*p* < 0.05). Ca changes varied significantly depending on the type of herb used. Herb-free groups (X) had the lowest Ca levels among all groups (*p* < 0.05), but Ca loss decreased depending on the herb type. Moreover, Ca content increased in gravlax samples made with sweet basil compared to fresh fillets, clearly indicating that Ca transferred from sweet basil to fish meat. Sage (S) groups exhibited the lowest Ca loss, maintaining values closest to fresh fillets. In summary, Ca contents increased significantly only in sweet basil-infused gravlax samples, whereas the sage-infused groups closely matched the fresh fillet Ca levels (*p* < 0.05).

Arsenic (As) is a non-essential contaminant that is present in the environment through bioaccumulation or biomagnification, originating from both anthropogenic and natural sources, and is considered one of the most hazardous elements in the environment [49,50]. Nevertheless, there is currently no upper limit established for As in seafood because the vast majority of arsenic detected in these products is of organic origin, which is considered to have minimal adverse health effects [50]. According to the latest data reported by the US Environmental Protection Agency (EPA), the daily intake of inorganic As (iAs) should not exceed 0.0003 mg/kg body weight per day [51]. For an individual weighing 80 kg, the maximum safe daily dose is calculated as 0.024 mg. Given that a serving of gravlax is 57 g [6], and considering that most of the As present in seafood is of organic origin, it can be stated that the gravlax produced in this study is safe for consumption with respect to As content. The primary concern here is the risk posed by inorganic arsenic. In both fresh fish fillets and gravlax groups, As concentrations were found to range between 0.03 and 0.06 mg/100 g. Considering that nearly all of the arsenic in fish meat is organic, and that the detected levels are already quite low, it can be concluded that As does not pose a health risk in this context.

Lead (Pb), as one of the major outcomes of environmental pollution, can enter aquatic environments and accumulate in fish muscle tissue. In particular, inorganic lead (iPb), even at low doses, can result in chronic accumulation in the human body, eventually causing damage to the nervous, circulatory, cardiovascular, bone, and cartilage systems, and is classified as a Group 2A carcinogen [52,53,54]. Commission Regulation (EC) No. 1881 has established the maximum permissible Pb level in fish meat at 0.03 mg/100 g [55]. The Pb levels detected in both gravlax and fresh fillet samples ranged from 0.00 to 0.01 mg/100 g, which is well below the legal limits.

Aluminum (Al) was detected in fresh garfish and tub gurnard fillets at concentrations of 0.08 and 0.20 mg/100 g, respectively. Slight increases in Al content were observed in gravlax samples due to the addition of herbs. The European Food Safety Authority (EFSA) has set the weekly tolerable intake for Al at 1 mg/kg body weight [56]. Accordingly, the maximum daily dose for an adult weighing 80 kg is 11.40 mg. The amount of Al taken in with the consumption of a single serving of gravlax (57 g) is therefore far below this limit. Silicon (Si) naturally occurs in human bodies and seafood, and most dietary Si is excreted without being absorbed, thus posing no toxic accumulation risk from a food safety perspective and is not limited [57]. Detected Si levels in gravlax samples were close to those of fresh fillets. Iron (Fe) levels increased slightly in the dill and basil-infused groups, whereas the other groups showed minimal changes (Table 3). Zinc (Zn) levels showed a slight decrease compared to fresh fillets. All other trace elements and heavy metals were detected at very low concentrations.

## 4. Conclusions

The results indicate that fish species and herb type significantly impact the physicochemical and sensory properties of gravlax. Color parameters notably varied with herbs, especially purple basil and sweet basil, creating perceptible visual distinctions. Moisture content and water activity decreased across all groups due to curing, resulting in proportional increases in protein and energy, particularly pronounced in garfish gravlax, where protein content reached 27%. Salt absorption was highest in herb-free gravlax but lowest in mint-containing groups, which contributed positively to texture and sensory acceptance. Mint and dill gravlax (particularly in the GM and SD groups) received consistently high sensory scores, indicating a strong consumer preference driven by pleasant firmness, mouthfeel, and overall acceptability. Conversely, sage-infused gravlax was least favored due to overpowering herb-salt interactions diminishing fish flavors.

Amino acid analysis revealed the highest total free amino acids, particularly umami and sweet-tasting compounds, in herb-free gravlax, suggesting herbs slow proteolytic degradation. Strong correlations between instrumental umami measurements and sensory perceptions (r > 0.90) emphasized targeted herb selection’s potential in enhancing desirable flavors. Sensory ratings for sweetness and saltiness aligned closely with instrumental data, affirming the reliability of consumer perceptions. The preference for mint and dill, combined with the visual and textural benefits of sweet basil and purple basil, highlights opportunities for product diversification tailored to specific market segments. This study underscores the value of integrating sensory evaluation with analytical data in developing innovative gravlax products, emphasizing the potential for further refinement in herb selection, curing conditions, and fish-herb interactions to enhance nutritional and sensory qualities. The impact of storage and shelf-life on product quality was not assessed in this research; however, further studies are planned to investigate these aspects in the future.

## Figures and Tables

**Table 1:** Formation and nomenclature of groups in the study.

Base Materials	Groups	Abbreviations	Explanations
Garfish (*Belone belone* Linnaeus, 1761) (Groups that are produced with garfish start with “G”)	Fillet	GF	Fresh, non-processed garfish fillet
	Gravlax with only salt, sugar and spices	GX	Garfish gravlax without any herbs
	Gravlax made with sage	GS	Garfish gravlax made with sage
	Gravlax made with dill	GD	Garfish gravlax made with dill
	Gravlax made with sweet basil	GSB	Garfish gravlax made with sweet basil
	Gravlax made with mint	GM	Garfish gravlax made with mint
	Gravlax made with purple basil	GPB	Garfish gravlax made with purple basil
Tub gurnard (*Chelidonichthys lucerna* Linnaeus, 1758) (Groups that are produced with tub gurnard start with “S”)	Fillet	SF	Fresh, non-processed tub gurnard fillet
	Gravlax with only salt, sugar and spices	SX	Tub gurnard gravlax without any herbs
	Gravlax made with sage	SS	Tub gurnard gravlax made with sage
	Gravlax made with dill	SD	Tub gurnard gravlax made with dill
	Gravlax made with sweet basil	SSB	Tub gurnard gravlax made with sweet basil
	Gravlax made with mint	SM	Tub gurnard gravlax made with mint
	Gravlax made with purple basil	SPB	Tub gurnard gravlax made with purple basil
Sage (*S. officinalis*)	Herbs	S	Analyses conducted solely on the herbs themselves (e.g., color, aw, pH, and sensory analyses) are denoted by the corresponding abbreviation, which also forms the last one or two letters of the gravlax group abbreviations where they are used.
Dill (*A. graveolens*)		D	
Sweet basil (*O. basilicum*)		SB	
Mint (*M. piperita*)		M	
Purple basil (*O. basilicum* var. *purpurascens*)		PB	

**Table 2:** Principal component analysis (PCA) matrix of amino acid parameters.

Amino Acids	PC 1	PC 2	Explanation
Ala	0.270	0.065	Both PC1 and PC2 exhibit positive loadings; however, the PC1 component is more dominant.
Thr	0.267	0.028	
Glu	0.267	0.027	
Tyr	0.263	0.024	
Pro	0.257	0.083	
Met	0.257	0.039	
Gly	0.203	0.041	
Arg	0.266	−0.115	PC1 exhibits a positive loading, while PC2 shows a negative loading; however, the influence of PC2 remains at a low level.
Lys	0.262	−0.096	
Asp	0.257	−0.067	
Leu	0.265	−0.066	
Phe	0.269	−0.018	
Ser	0.250	0.223	Both principal components exhibit positive loadings in a similar direction.
Orn	0.071	0.050	The least influential variable in both principal components.
Cys	0.121	0.501	The variables with the most substantial positive loadings on PC2
His	0.169	0.486	
Val	0.203	−0.483	The variables with the most substantial negative loadings on PC2.
Ile	0.206	−0.422	

**Table 3:** Mineral substances and heavy metals of gravlax groups and fresh fillets (mg/100 g).

		GF	GX	GS	GD	GSB	GM	GPB	SF	SX	SS	SD	SSB	SM	SPB
Major minerals	Ca	270.40 ± 2.05 ^c^	70.74 ± 1.46 ^g^	217.19 ± 3.22 ^d^	140.58 ± 1.52 ^f^	297.37 ± 2.05 ^b^	135.35 ± 0.45 ^f^	144.07 ± 3.14 ^f^	225.04 ± 0.15 ^d^	20.69 ± 0.24 ^j^	181.67 ± 0.24 ^e^	30.73 ± 0.20 ^I, j^	326.46 ± 2.60 ^a^	33.95 ± 0.88 ^i^	45.00 ± 0.20 ^h^
	Mg	59.17 ± 0.56 ^d^	92.42 ± 0.34 ^a^	64.20 ± 0.25 ^b^	54.93 ± 0.29 ^e^	53.86 ± 0.25 ^e, f^	60.52 ± 0.34 ^c, d^	52.49 ± 0.15 ^f^	46.55 ± 0.27 ^h^	52.53 ± 0.09 ^f^	46.48 ± 0.09 ^h^	41.30 ± 0.52 ^i^	49.87 ± 0.17 ^g^	60.99 ± 0.13 ^c^	46.36 ± 0.40 ^h^
	Na	276.14 ± 4.05 ^k^	7654.75 ± 11.99 ^a^	5723.93 ± 3.35 ^e^	6386.11 ± 14.39 ^b^	5209.19 ± 3.35 ^f^	5052.90 ± 18.30 ^g^	6083.08 ± 26.74 ^c^	312.42 ± 8.37 ^k^	5843.29 ± 22.75 ^d^	4050.36 ± 28.31 ^j^	4994.63 ± 28.31 ^h^	5677.88 ± 16.48 ^e^	4104.70 ± 24.04 ^j^	4582.11 ± 57.76 ^i^
	K	638.88 ± 2.67 ^a^	527.51 ± 4.34 ^d^	470.51 ± 1.54 ^e^	435.23 ± 2.59 ^f^	437.55 ± 1.54 ^f^	521.74 ± 1.81 ^d^	441.95 ± 1.06 ^f^	644.27 ± 4.46 ^a^	549.25 ± 2.88 ^c^	574.70 ± 2.88 ^b^	510.95 ± 2.62 ^d^	521.69 ± 5.38 ^d^	550.57 ± 11.03 ^c^	533.49 ± 3.12 ^c, d^
	P	580.79 ± 4.11 ^a^	529.17 ± 3.74 ^b^	352.93 ± 5.84 ^f, g^	434.99 ± 2.51 ^e^	485.04 ± 1.67 ^d^	465.35 ± 2.68 ^d^	422.42 ± 5.04 ^e^	565.46 ± 1.67 ^a, b^	425.54 ± 1.87 ^e^	367.53 ± 1.87 ^f^	309.20 ± 3.25 ^h^	504.52 ± 7.33 ^c^	341.32 ± 4.62 ^g^	313.82 ± 1.52 ^h^
Essential minerals	Cu	0.07 ± 0.00 ^d^	0.13 ± 0.00 ^a^	0.08 ± 0.00 ^c, d^	0.06 ± 0.00 ^e^	0.08 ± 0.00 ^c, d^	0.07 ± 0.00 ^d^	0.08 ± 0.00 ^c^	0.04 ± 0.00 ^g, h^	0.10 ± 0.00 ^b^	0.05 ± 0.00 ^f^	0.05 ± 0.00 ^f, g^	0.04 ± 0.00 ^g, h^	0.05 ± 0.00 ^f^	0.03 ± 0.00 ^h^
	Fe	0.74 ± 0.00 ^e, f^	0.75 ± 0.00 ^e^	0.70 ± 0.00 ^g^	1.16 ± 0.01 ^b^	1.26 ± 0.00 ^a^	0.73 ± 0.00 ^e, f^	0.76 ± 0.01 ^d^	0.51 ± 0.00 ^h^	0.45 ± 0.00 ^i^	0.71 ± 0.00 ^f, g^	0.90 ± 0.00 ^c^	0.90 ± 0.00 ^c^	0.50 ± 0.00 ^h^	0.52 ± 0.00 ^h^
	Mn	0.06 ± 0.00 ^i, j^	0.04 ± 0.00 ^j^	0.26 ± 0.00 ^b^	0.24 ± 0.00 ^c^	0.22 ± 0.00 ^d^	0.14 ± 0.00 ^g^	0.12 ± 0.00 ^h^	0.04 ± 0.00 ^j^	0.06 ± 0.00 ^i^	0.19 ± 0.00 ^e^	0.16 ± 0.00 ^f^	0.36 ± 0.00 ^a^	0.13 ± 0.00 ^g, h^	0.20 ± 0.00 ^e^
	Se	0.07 ± 0.00 ^d^	0.12 ± 0.00 ^a^	0.07 ± 0.00 ^d^	0.07 ± 0.00 ^d^	0.07 ± 0.00 ^d^	0.07 ± 0.00 ^d^	0.06 ± 0.00 ^d^	0.11 ± 0.01 ^a, b^	0.09 ± 0.00 ^c^	0.09 ± 0.00 ^c^	0.10 ± 0.00 ^b, c^	0.09 ± 0.00 ^c^	0.09 ± 0.00 ^b, c^	0.09 ± 0.00 ^c^
	Zn	3.16 ± 0.04 ^a^	2.19 ± 0.01 ^e^	2.48 ± 0.02 ^c, d^	2.61 ± 0.04 ^c^	2.90 ± 0.02 ^b^	2.41 ± 0.02 ^d^	2.50 ± 0.03 ^c, d^	0.84 ± 0.00 ^f^	0.69 ± 0.00 ^f, g^	0.69 ± 0.01 ^f, g^	0.59 ± 0.01 ^g, h^	0.66 ± 0.00 ^g^	0.71 ± 0.01 ^f, g^	0.49 ± 0.01 ^h^
Heavy metals	Al	0.08 ± 0.00 ^h^	0.19 ± 0.00 ^d^	0.31 ± 0.00 ^a^	0.29 ± 0.00 ^b^	0.29 ± 0.00 ^b^	0.18 ± 0.00 ^e^	0.15 ± 0.00 ^g^	0.20 ± 0.00 ^d^	0.15 ± 0.00 ^g^	0.24 ± 0.00 ^c^	0.20 ± 0.00 ^d^	0.30 ± 0.00 ^a, b^	0.17 ± 0.00 ^f^	0.29 ± 0.00 ^b^
	As	0.04 ± 0.00 ^d, e, f^	0.06 ± 0.00 ^a^	0.05 ± 0.00 ^b^	0.04 ± 0.00 ^c, d, e^	0.06 ± 0.00 ^a^	0.04 ± 0.00 ^e, f^	0.05 ± 0.00 ^b, c^	0.04 ± 0.00 ^d, e, f^	0.04 ± 0.00 ^c, d, e^	0.04 ± 0.00 ^f^	0.05 ± 0.00 ^b, c^	0.04 ± 0.00 ^b, c, d^	0.04 ± 0.00 ^f^	0.03 ± 0.00 ^g^
	Pb	0.01 ± 0.00 ^a^	0.01 ± 0.00 ^b^	0.00 ± 0.00 ^e, f^	0.01 ± 0.00 ^d, e^	0.00 ± 0.00 ^e, f^	0.01 ± 0.00 ^c^	0.00 ± 0.00 ^f, g^	0.01 ± 0.00 ^b^	0.01 ± 0.00 ^a^	0.00 ± 0.00 ^f, g^	0.01 ± 0.00 ^c, d, e^	0.00 ± 0.00 ^f, g^	0.01 ± 0.00 ^c, d^	0.00 ± 0.00 ^g^
Others	B	0.12 ± 0.00 ^a^	0.08 ± 0.00 ^c^	0.06 ± 0.00 ^e^	0.07 ± 0.00 ^d^	0.06 ± 0.00 ^f, g^	0.05 ± 0.00 ^h^	0.04 ± 0.00 ^i^	0.07 ± 0.00 ^d^	0.05 ± 0.00 ^i^	0.06 ± 0.00 ^e, f^	0.06 ± 0.00 ^e, f^	0.05 ± 0.00 ^g, h^	0.09 ± 0.00 ^b^	0.06 ± 0.00 ^e^
	Ba	0.01 ± 0.00 ^g^	0.01 ± 0.00 ^g^	0.03 ± 0.00 ^b^	0.03 ± 0.00 ^c^	0.03 ± 0.00 ^b^	0.02 ± 0.00 ^f^	0.02 ± 0.00 ^e, f^	0.01 ± 0.00 ^g^	0.01 ± 0.00 ^g^	0.02 ± 0.00 ^d, e^	0.02 ± 0.00 ^e, f^	0.04 ± 0.00 ^b^	0.04 ± 0.00 ^a^	0.02 ± 0.00 ^c, d^
	Ni	0.01 ± 0.00 ^d^	0.04 ± 0.00 ^b^	0.01 ± 0.00 ^e, f^	0.01 ± 0.00 ^f, g^	0.00 ± 0.00 ^g^	0.00 ± 0.00 ^g^	0.00 ± 0.00 ^g^	0.03 ± 0.00 ^c^	0.05 ± 0.00 ^a^	0.01 ± 0.00 ^e^	0.01 ± 0.00 ^d^	0.00 ± 0.00 ^g^	0.01 ± 0.00 ^f, g^	0.00 ± 0.00 ^g^
	Rb	0.07 ± 0.01 ^f^	0.12 ± 0.00 ^a, b^	0.07 ± 0.00 ^e, f^	0.08 ± 0.00 ^e, f^	0.08 ± 0.00 ^d, e, f^	0.09 ± 0.00 ^d, e, f^	0.08 ± 0.00 ^e f^	0.10 ± 0.00 ^b, c, d^	0.14 ± 0.00 ^a^	0.10 ± 0.00 ^b, c, d, e^	0.09 ± 0.00 ^c, d, e, f^	0.10 ± 0.00 ^b, c, d, e^	0.11 ± 0.01 ^b, c^	0.10 ± 0.00 ^b, c, d, e^
	Si	2.67 ± 0.00 ^b^	2.72 ± 0.00 ^a^	2.71 ± 0.01 ^a^	2.50 ± 0.00 ^d^	2.62 ± 0.01 ^c^	2.13 ± 0.00 ^g^	2.04 ± 0.00 ^h^	2.73 ± 0.01 ^a^	2.71 ± 0.01 ^a^	2.70 ± 0.01 ^a^	2.51 ± 0.01 ^d^	2.59 ± 0.00 ^c^	2.18 ± 0.01 ^f^	2.37 ± 0.01 ^e^
	Sr	0.75 ± 0.01 ^c^	0.24 ± 0.00 ^f^	0.84 ± 0.01 ^b^	0.47 ± 0.00 ^d^	0.85 ± 0.01 ^b^	0.42 ± 0.00 ^e^	0.45 ± 0.01 ^d^	0.09 ± 0.00 ^i^	0.06 ± 0.00 ^j^	0.19 ± 0.00 ^g^	0.13 ± 0.00 ^h^	0.93 ± 0.00 ^a^	0.19 ± 0.00 ^g^	0.19 ± 0.00 ^g^
	Ti	0.07 ± 0.00 ^e, f, g^	0.12 ± 0.01 ^a, b^	0.12 ± 0.00 ^a^	0.08 ± 0.00 ^e, f, g^	0.09 ± 0.00 ^c, d^	0.08 ± 0.00 ^e, f, g^	0.08 ± 0.00 ^d, e, f^	0.07 ± 0.00 ^f, g^	0.07 ± 0.00 ^g^	0.09 ± 0.00 ^d, e^	0.08 ± 0.00 ^e, f, g^	0.11 ± 0.00 ^b, c^	0.09 ± 0.00 ^d, e^	0.08 ± 0.00 ^e, f, g^

(a, b, c, d, e, f, g, h, i, j, k) Means with different lowercase letters in the same row are significantly different (*p* < 0.05).

## Data Availability

The original contributions presented in the research are included in the article/Supplementary Materials, further inquiries can be directed to the corresponding author.

## Supplementary Materials

The following supporting information can be downloaded at https://www.mdpi.com/article/10.3390/foods14142465/s1, Table S1: The sensory survey of herb groups using a 9-point scale.; Table S2: The sensory survey of gravlax groups using a 9-point and triple-*X* scale.
