# Supplementary material for: A Comprehensive Study on Gravlax: A Multidimensional Evaluation of Gravlax Produced from Different Fish Species and Herbs
Source: Foods. 2025 Jul 14;14(14):2465. doi: 10.3390/foods14142465 (PMC12294933; doi:10.3390/foods14142465)
Supplement: Supplementary file 1 [file foods-14-02465-s001.zip › Table_S1.pdf]

Table S1. The sensory survey of herb groups using a 9-point scale.

Score	Preliminary Opinion on the Herb's Suitability for Gravlax	Mouthfeel During Consumption	Mouthfeel After Consumption	Aroma Liking	Sourness Liking	Visual Appeal	Overall Appeal	Willingness to Use the Herb Differently (e.g. drying, brewing) or Add to the Foods After Taste	Favorite Herb
**9**	Extremely suitable	Like extremely						Extremely willing	1st choice
**8**	Very suitable	Like very much						Very willing	2nd choice
**7**	Suitable	Like moderately						Willing	3rd choice
**6**	Slightly suitable	Like slightly						Slightly willing	4th choice
**5**	Moderately suitable	Neither like nor dislike						Moderately willing	5th choice
**4**	Slightly unsuitable	Dislike slightly						Slightly unwilling	6th choice
**3**	Moderately unsuitable	Dislike moderately						Moderately unwilling	7th choice
**2**	Very unsuitable	Dislike very much						Very unwilling	8th choice
**1**	Not at all suitable	Dislike extremely						Not at all willing	9th choice
Survey Responses Section (Please score the following boxes with a scale of 1 to 9)									
	**Preliminary Opinion on the Herb's Suitability for Gravlax**	**Mouthfeel During Consumption**	**Mouthfeel After Consumption**	**Aroma Liking**	**Sourness Liking**	**Visual Appeal**	**Overall Appeal**	**Willingness to Use the Herb Differently (drying, brewing, etc) or Add to the Foods After Taste**	**Favorite Herb**
**Dill (D)**									
**Mint (M)**									
**Sage (S)**									
**Sweet Basil (SB)**									
**Purple Basil (PB)**									
Please write your comments, suggestions, and criticisms about the herbs in detail in this section.									
