# Supplementary material for: A Comprehensive Study on Gravlax: A Multidimensional Evaluation of Gravlax Produced from Different Fish Species and Herbs
Source: Foods. 2025 Jul 14;14(14):2465. doi: 10.3390/foods14142465 (PMC12294933; doi:10.3390/foods14142465)
Supplement: Supplementary file 1 [file foods-14-02465-s001.zip › Table_S2.pdf]

Table S2. The sensory survey of gravlax groups using a 9-point and triple-*X* scale.

Score	***Appearance***	***Texture***	***Sweetness Liking***	***Saltiness Liking***	***Sourness Liking***	***Aroma Liking***	***Umami Feeling***	***General Acceptability***	***Favorite Gravlax***
9	Extremely appealing	Extremely desirable	Ideal sweetness, perfectly balanced	Ideal saltiness, perfectly balanced	Ideal sourness, perfectly balanced	Extremely dense, very intense	Extremely intense umami	Extremely acceptable	Please answer this question independently from all other questions. Upon your first tasting, did you feel that "I really liked that gravlax, and I would like to eat it again in the future"?<br><br>(Among the 10 types of gravlax, please mark only your top 3 favorites in order with '*XXX*', '*XX*', and '*X*,' respectively. Please leave the other boxes blank.)
8	Very appealing	Very desirable	In near-ideal balance, very pleasant	In near-ideal balance, very pleasant	In near-ideal balance, very pleasant	Very dense, clearly perceptible	Very intense umami	Very acceptable	
7	Moderately appealing	Moderately desirable	Clearly perceptible sweetness, pleasant	Clearly perceptible saltiness, pleasant	Clearly perceptible sourness, pleasant	Moderately dense, clearly noticeable	Moderately intense umami	Moderately acceptable	
6	Slightly appealing	Slightly desirable	Mildly perceptible sweetness	Mildly perceptible saltiness	Mildly perceptible sourness	Slightly dense, mildly perceptible	Slightly perceptible umami	Slightly acceptable	
5	Neutral	Neutral texture, acceptable	Barely perceptible sweetness	Barely perceptible saltiness	Barely perceptible sourness	Neutral aroma density, mild	Barely perceptible umami	Neither acceptable nor unacceptable	
4	Slightly unappealing	Slightly undesirable	Slightly lacking sweetness	Slightly lacking saltiness	Slightly lacking sourness	Slightly weak aroma density	Slightly lacking umami	Slightly unacceptable	
3	Moderately unappealing	Moderately undesirable	Noticeably lacking sweetness	Noticeably lacking saltiness	Noticeably lacking sourness	Moderately weak aroma density	Noticeably lacking umami	Moderately unacceptable	
2	Very unappealing	Very undesirable	Barely detectable sweetness, insufficient	Barely detectable saltiness, insufficient	Barely detectable sourness, insufficient	Very weak aroma density	Very weak umami, almost undetectable	Very unacceptable	
1	Extremely unappealing	Extremely undesirable	No sweetness or extreme sweetness	No saltiness or extreme saltiness	No sourness or extreme sourness	Aroma is not perceptible	No umami or extreme umami feeling	Extremely unacceptable	

Survey Responses Section (Please score the following boxes with a scale of 1 to 9, and please mark only your top 3 favorites in order with 'XXX', 'XX', and 'X' in the last column)

	Appearance	***Texture***	***Sweetness Liking***	***Saltiness Liking***	***Sourness Liking***	***Aroma Liking***	***Umami feeling***	***General acceptability***	***Favorite gravlax***
SD									
SM									
SS									
SSB									
SPB									
GD									
GM									
GS									
GSB									
GPB									

Please write your comments, suggestions, and criticisms about the gravlaxs in detail in this section.
